# Cheminformatics Study on Structural and Bactericidal Activity of Latest Generation β-Lactams on Widespread Pathogens

**DOI:** 10.3390/ijms232012685

**Published:** 2022-10-21

**Authors:** Ana Maria Raluca Gherman, Nicoleta Elena Dina, Vasile Chiș

**Affiliations:** 1 Department of Molecular and Biomolecular Physics, National Institute for R&D of Isotopic and Molecular Technologies, Donat 67-103, 400293 Cluj-Napoca, Romania; 2 Faculty of Physics, Babeș-Bolyai University, Kogălniceanu 1, 400084 Cluj-Napoca, Romania

**Keywords:** azlocillin, carbenicillin, oxacillin, molecular docking, antibiotic susceptibility testing, β-lactams, Raman spectrum, density functional theory

## Abstract

Raman spectra of oxacillin (OXN), carbenicillin (CBC), and azlocillin (AZL) are reported for the first time together with their full assignment of the normal modes, as calculated using Density Functional Theory (DFT) methods with the B3LYP exchange-correlation functional coupled to the 6-31G(d) and 6-311+G(2d,p) basis sets. Molecular docking studies were performed on five penicillins, including OXN, CBC, and AZL. Subsequently, their chemical reactivity and correlated efficiency towards specific pathogenic strains were revealed by combining frontier molecular orbital (FMO) data with molecular electrostatic potential (MEP) surfaces. Their bactericidal activity was tested and confirmed on a couple of species, both Gram-positive and Gram-negative, by using the disk diffusion method. Additionally, a surface-enhanced Raman spectroscopy (SERS)—principal component analysis (PCA)-based *resistogram* of *A. hydrophila* is proposed as a clinically relevant insight resulting from the synergistic cheminformatics and vibrational study on CBC and AZL.

## 1. Introduction

Antibiotics are drugs with specific activity against bacteria, acting by either killing the pathogen or inhibiting its growth. The inhibition takes place by interfering with the process of cell-wall synthesis, a specific mechanism of action to the group of antibiotics called *β-lactams*. Basically, *β-lactams* target the *penicillin-binding protein (PBP)* enzymes and irreversibly inhibit them, hindering biosynthesis of the peptidoglycan layer of the bacterial cell wall. By compromising the cell wall’s integrity, cell autolysis is caused. Of the several classes of *β-lactams*, penicillins are the most diverse and most commonly used.

Penicillins are a group of natural antibacterials semisynthetized from *Penicillium* mold, with a general molecular formula of R-C_9_H_11_N_2_O_4_S. A penicillin molecule is made up of two main components: A *penam core* common for all penicillins—a 6-aminopenicillanic acid (6-APA)—and a side-chain R, which varies from one penicillin to another. 6-APA (shown in Table 1) is the starting block of the synthesis and is obtained naturally from the fermentation medium of the mold. It consists of a *thiazolidine ring* (a five-membered saturated ring with a thioether group in position 1 and an amine group in position 4) merged with 2-azetidinone (a four-membered *β-lactam*). The various R side-chains from the 6-position of 6-APA are the ones that make a difference in penicillin’s molecular structure. The final products are the synthesis results of fermentation to which particular side-chains are added [1].

*Benzylpenicillin (BPN),* or penicillin G, is natural penicillin (Table 1) with the simplest chemical structure of them all. Its particularity is the phenylacetamido side chain at the 6-position of 6-APA. With the aid of DFT calculations, the electronic and chemical structure of BPN was intensively studied, including the full assignments of the normal modes, in combination with vibrational spectroscopies [2,3]. Moreover, its adsorption on both Au and Ag substrates was also elucidated by the SERS technique [2,4,5,6]. *Oxacillin (OXN)* is a β-lactamase resistant penicillin (Table 1).

*Ampicillin (APN)* is a member of the aminopenicillin family (third generation). The chemical structure differs from BPN only by having an extra amino group on its side chain (a D(-)-α-aminophenylacetamido side-chain), which actually serves to penetrate the outer membrane of Gram-negative (GN) bacteria (Table 1). Being the first penicillin with bactericidal activity against GN bacteria including *Escherichia coli*, *Enterococcus* spp, and several *Staphylococcus* spp., numerous studies on APN’s electronic and chemical structure have been reported to date, using vibrational spectroscopies (FT-IR, FT-Raman, Raman, SERS) and DFT calculations. These studies reported various polymorphic forms of APN [7,8], its monomer [9,10], dimer, or tetramer [11]. Computational techniques were employed for characterizing, from the electronic structure and physicochemical properties points of view, different penicillins, among which were BPN, APN, and CBC [12,13], as well as a *molecular docking* scanning [10], in order to elucidate their biological action. Numerical simulations on gold/ampicillin systems resulting in adsorption energies, electron densities, and bond distances, as well as ampicillin’s orientation relative to the gold surface, are also reported [14]. Another study using SERS reported the detection and quantification of APN, BPN, and CBC in the presence of their degradation products [15].

*Carbenicillin (CBC)*, fourth-generation penicillin, is a semi-synthetic precursor of BPN, similar to APN. It belongs to the carboxypenicillin subgroup, having an α-carboxyphenylacetamido side-chain. Basically, the amido group from ampicillin’s side-chain is replaced by a carboxyl group on the CBC’s side-chain (Table 1).

*Azlocillin (AZL)* is a member of the ureidopenicillins (fifth generation). They derive from APN, in which different cyclic ureas are added to the amino group on the side-chain of the molecule (Table 1). Its side-chain imitates a segment of the peptidoglycan chain better than APN, thus AZL can bind more easily to the PBPs.

Penicillins are still in use, and newer strategies are employed for their boosted efficiency: Synergy with peptides as adjuvants in the case of AMP and OXN (efficient upward 2 h testing timeline) [16] or by managing their resistance breakpoints, since there are already OXN-resistant superbugs (OR-MRSA) [17]. For OXN, the determined *minimum inhibitory concentration (MIC)* is ≤2 µg/mL for the sensitivity case (S) and ≥4 µg/mL for the resistance case (R) [16,17]. Another modern approach to boosted antibiotic therapy is the design of positively charged cyclodextrin hosts with enhanced binding of penicillins as carriers for their delivery [18]. In this case, by using a promising cyclodextrin-based vehicle (γCys) for the protection and delivery of OXN, its specific β-lactamase hydrolysis rate was considerably reduced. This is considered a suitable solution when antibiotic options in the treatment of extended-spectrum β-lactamases (ESBL)-producing organisms are limited [19].

Current vibrational studies employ normal Raman spectroscopy and SERS to partially characterize and detect benzylpenicillin sodium [6], carbenicillin disodium [13], or AZL [20] with high sensitivity. A comprehensive vibrational and cheminformatics study regarding the antimicrobial efficiency and the antibiotics’ mechanisms of action at the bacterial cell wall is achieved by combining analytical techniques with computational tools. This way we are able to calculate, for each penicillin candidate, their specific chemical reactivity and estimate their practical bactericidal potential.

SERS combines the inherent molecular specificity of the Raman technique and its obliviousness to water, providing a trace-level detection potential [13,20] enabled by the plasmonic properties of nanostructured colloidal [13] or nanopatterned solid films systems [21]. In biological applications targeting cellular viability [22] or drug susceptibility [23], silver-based nanoparticles (AgNPs) render high sensitivity and reproducibility due to their particular optical properties [24] even when used in most simple approaches [25,26,27]. SERS is currently used as an alternative in obtaining fast *antibiotic susceptibility testing (AST)* for common [28] or multi-resistant pathogens [29,30,31], in already numerous promising research studies [32,33,34]. Real-time SERS measurements for in vitro monitoring of microbial cultures undergoing susceptibility tests toward antibiotics are now possible with reliable results. The ultrasensitivity of SERS-based detection and its suitability in monitoring viable, biological samples are the key aspects explored in clinical applications [35].

Thus, herein, we explored the chemical reactivity and bactericidal properties of a series of penicillins, one for each of the first five generations, primarily by shedding light on the structural differences of their ground-state optimized structures. This was achieved by providing a full assignment of the normal modes observed by Raman spectroscopy, and computing the MEP surfaces, the energy gap between the highest occupied molecular orbital (HOMO) and the lowest unoccupied molecular orbital (LUMO), and chemical reactivity descriptors. This synergistic approach enables us to gain extensive insight into how small molecules interact with their targets, especially when designing new drugs [36,37,38], and, in our case, how their bactericidal activity varies from one generation to another, particularly in how the antibiotics bind to macromolecular targets in the bacterial cell-wall. An up-to-date molecular docking study was performed on several proteins found in the bacterial cell wall to better understand the conformations adopted by the antibiotics within the binding sites of these macromolecules and the nature of these chemical interactions. The penicillins were also evaluated against three GP and three GN strains in support of the in silico study. Additional insight regarding their practical efficiency was gained by monitoring the specific SERS profiles of bacterial cells undergoing antibiotic stress, or control conditions, respectively.

The novelty of this complex investigation is that both in silico and experimental protocols were unified in one *resistogram*-like result concerning the mechanism of action for five penicillins on widespread microbial pathogens with the final scope of selecting the most promising candidate in practice. In addition, AZL is characterized for the first time from a vibrational and computational point of view. Quantum chemical reactivity descriptors and the key outcomes obtained from in silico calculations revealed AZL as a suitable candidate in the case of OXN-resistant species, showing similar efficiency to OXN against PBP responsible for widespread infections. This represents an important aspect in practice, when *a priori* in silico studies on the most relevant pathogens and corresponding recommended antibiotics could provide a fast drug *susceptibility* output without the actual need for tedious cultivation of microbial species.

## 2. Results and Discussion

### 2.1. Chemical Structure (Dis)Similarities Observed in the Vibrational Spectra

**Figure 1:**
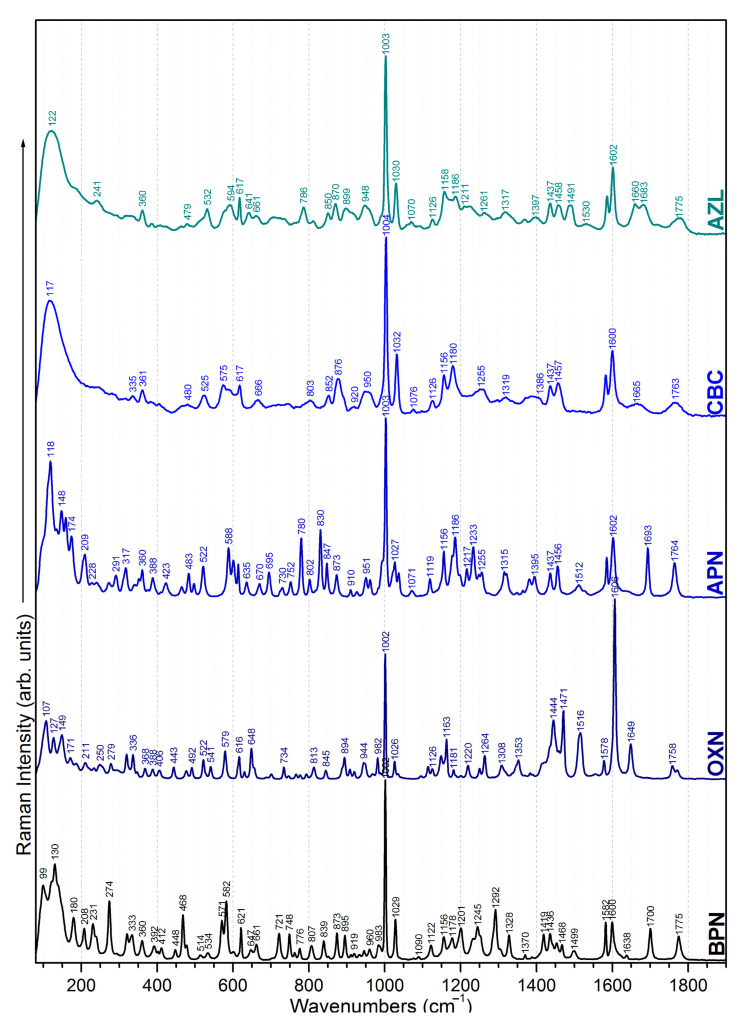
FT-Raman (laser line 1064 nm) spectrum of BPN, OXN, APN, AZL, and CBC.

Since all five considered antibiotics belong to the penicillin family, most of their chemical structures are similar. As seen in Table 1, the main chemical groups common to all five antibiotics examined in this study are the benzene ring, two methyl and two carbonyl groups, a carboxyl and an amide group, and lastly, a *thiazolidine* and a *β-lactam ring*. As for the dissimilarities, we list them by increasing the class they belong to: Two H atoms in the CH2 group next to the benzene ring for BPN; an isoxazole ring for OXN; an amino group for the APN; an additional carboxyl group for CBC, which replaces the amino group of APN; and lastly, an amide group, two carboxyl groups, and an imidazolidine ring for AZL, replacing the same amino group of APN. We assigned the normal modes based on frequency calculations performed by DFT calculations. Full assignments of Raman spectra for BPN, OXN, APN, CBC, and AZL are listed in Table S1 together with calculated Raman bands at harmonic and anharmonic levels. The FT-Raman spectrum of each molecule is also plotted in Figure 1. Further, we will discuss Raman marker bands of penicillins (i.e., common to all five antibiotics), and the specific vibrational modes of each antibiotic, listed in Table 2.

#### 2.1.1. Common Bands

The band with the strongest intensity for all five molecules at 1002–1004 cm^−1^ corresponds to an in-plane deformation of the **benzene ring**. Other contributions from the benzene ring can be observed as the weak band at 615–621 cm^−1^ due to the in-plane deformation of the ring with an in-plane bending of its CH groups. Bending of CH groups is assigned to the 830–845 cm^−1^, 957–993 cm^−1^, and 1026–1032 cm^−1^ bands as well. Furthermore, 1578–1585 cm^−1^ and 1600–1606 cm^−1^ correspond to CC stretching combined with CH bending vibrations. This doublet has different intensity ratios from one antibiotic to another—both weak for BPN, and very weak and medium for APN, CBC, and AZL. For OXN, 1602 cm^−1^ is actually the strongest band in the whole spectrum. This is because, in this case, the benzene ring forms a bond with another ring (isoxazole ring) rather than a simple C atom as with the other antibiotics.

The **two methyl groups** common to all five considered penicillins are Raman active and can be observed in all spectra at 231–250 cm^−1^ and 271–280 cm^−1^ as CH_3_ rocking normal mode; CH_3_ wagging is assigned to the bands at 291–296 cm^−1^ and 948–960 cm^−1^; whereas 1433–1437 cm^−1^ and 1452–1459 cm^−1^ are due to the CH_3_ bending mode.

The most intense Raman bands assigned to OH bending from the **carboxyl group** are the doublets observed at 870–876 cm^−1^ and 894–910 cm^−1^. OH bending comes as a second contribution to these bands. We assigned OH bending to the bands at 522–525 cm^−1^, 802–807 cm^−1^, and 1245–1249 cm^−1^. While 1245–1249 cm^−1^ is actually a combination of OH stretching and bending, OCO bending is assigned for 802–807 cm^−1^ as well. The OCO chemical group presents another two Raman responses, as a rocking normal mode at 360–268 cm^−1^ and as a stretching normal mode at 919–920 cm^−1^.

The weak 656–670 cm^−1^ band is the Raman response for the out-of-plane bending of the N9H **amide group**, as well as 1175–1178 cm^−1^, where the normal mode comes as a second contribution.

The Raman response of the C8O23 **carbonyl group** consists of two bands: 402–409 cm^−1^ assigned to in-plane bending of the group and 1638–1693 cm^−1^ assigned to the stretching.

The bands at 571–579 cm^−1^ assigned to CCC and CCN bending, 601–616 cm^−1^ assigned to NCS bending, 919–926 cm^−1^ assigned to CNC bending, and 1292–1297 cm^−1^ assigned to out-of-plane bending of CH bonds are the Raman response of ***thiazolidine ring***.

The 944–945 cm^−1^ band is assigned to CC stretching and CH bending, the one at 1156–1158 cm^−1^ is assigned to out-of-plane bending of CH groups, whereas the one at 1758–1775 cm^−1^ corresponds to C=O stretching and OH bending, with all chemical groups belonging to the ***β-lactam ring***.

#### 2.1.2. Specific Bands

Normal modes assigned to **methylene group** CH_2_ specific to BPN can be observed at 468 cm^−1^ (bending of CH) and 1419 cm^−1^ (bending of CH_2_) in the spectrum with medium to weak intensity, while in the other four cases, there is no observed signal.

**OXN**’s isoxazole ring provides the largest variances in its Raman spectrum. In addition to the most intense band in the spectrum at 1602 cm^−1^, which for all the other antibiotics is the in-plane deformation of the benzene ring at 1002–1004 cm^−1^, the **isoxazole ring** contributes to another set of normal modes as follows: 250 cm^−1^ and 1444 cm^−1^ are assigned to the twisting and bending of the C28H_3_ methyl group attached to the ring; 336 cm^−1^ is assigned to benzene-isoxazole ring bending; 492 cm^−1^ is due to N24H bending; in-plane and out-of-plane deformation of the ring is present in the spectrum at 734 cm^−1^, and 648 cm^−1^ and 793 cm^−1^, respectively; stretching vibrations are assigned to the N24O25 bond at 908 cm^−1^, to C7=N24 and C26O25, at 1308 cm^−1^, and to C7=N24 and C26C27, at 1471 cm^−1^; the band at 1516 cm^−1^ has a second contribution to the C6C7 stretching normal mode, with a significantly increased intensity compared to its correspondents in the other four spectra; and the C26C27 stretching, at 1556 cm^−1^. Most of these bands are of weak and very weak intensity, except the medium 1444 cm^−1^ and 1471 cm^−1^ bands.

In **APN**’s Raman spectrum, the very weak 465 cm^−1^ and 1638 cm^−1^ are assigned to the bending mode, while 830 cm^−1^ and 1186 cm^−1^ are assigned to the twisting of the NH_2_ amino group. The Raman signal at 1119 cm^−1^ and 1512 cm^−1^ is assigned to the C7N24 stretching combined with N24H and C7H bending. Except for 830 cm^−1^, all bands have a very weak intensity.

The specific bands of **CBC** differ from **carboxyl** vibrational modes in the other four spectra mainly in intensity since CBC has two carboxyl groups in its structure, as compared to a single one for the rest. The OCO bending can be observed very weakly at 666 cm^−1^ and 746 cm^−1^, while the wagging is at 1126 cm^−1^. We assigned stretching of the carbonyl group (C24=O) to the weak bands at 1763 cm^−1^ and 1666 cm^−1^. C7C24 stretching is very weakly observed at 1362 cm^−1^ and 1372 cm^−1^, while the latter comes with an OH contribution as well. OH bending can also be observed at 1180 cm^−1^.

Specific bands in **AZL**’s Raman spectrum are related to vibrations of the **imidazolidine ring** and the N24H **amide group**. The normal modes of the imidazolidine ring are either an in-plane deformation of the ring, at 1125 cm^−1^; in-plane and out-of-plane bending of N30H, observed at 465 cm^−1^, 714 cm^−1^, and 958 cm^−1^. We assigned the rocking of CH_2_ as a second contribution to 715 cm^−1^, and CH_2_ wagging to 1239 cm^−1^ and 1396 cm^−1^. CC stretching is assigned as a second contribution to the 958 cm^−1^ band. Vibrations of the N24H **amide group** are Raman active at 641 cm^−1^ and 1532 cm^−1^, and we assigned them the out-of-plane and in-plane bending of the N42H group. All AZL-specific Raman responses are of weak intensity.

Due to their common chemical structure—a benzene, a thiazolidine, and a β-lactam ring, two methyl and two carbonyl groups, a carboxyl, and an amide group—in their structure, penicillins come with a specific Raman response, with 1002–1004 cm^−1^ being the most intense band in all spectra. The most intense of the methyl groups is the doublet 1433–1437/1452–1459 cm^−1^, whereas the doublet 870–876/894–895 cm^−1^ is the most intense response of the carboxyl group. The deformation of the thiazolidine ring can be observed at 571–579 cm^−1^, while the response of the β-lactam ring is the most intense at 1156–1158 cm^−1^ and 1158–1175 cm^−1^. Specific marker bands for each antibiotic—BPN, OXN, APN, CBC, and AZL—are present in their spectra, as expected, but most of the Raman response is subtle, having weak and very weak intensity. OXN’s Raman spectrum stands out the most, due to the increased intensity of 1606 cm^−1^ (C6C7 stretching), as well as 1444 cm^−1^ (C28H_3_ bending) and 1471 cm^−1^ (C7=N24 and C26C27 stretching). APN’s Raman spectrum comes with more visible differences as well—780 cm^−1^ (NH bending) and 830 cm^−1^ (NH_2_ twisting). 

### 2.2. Molecular Electrostatic Potentials (MEPs)

**Figure 2:**
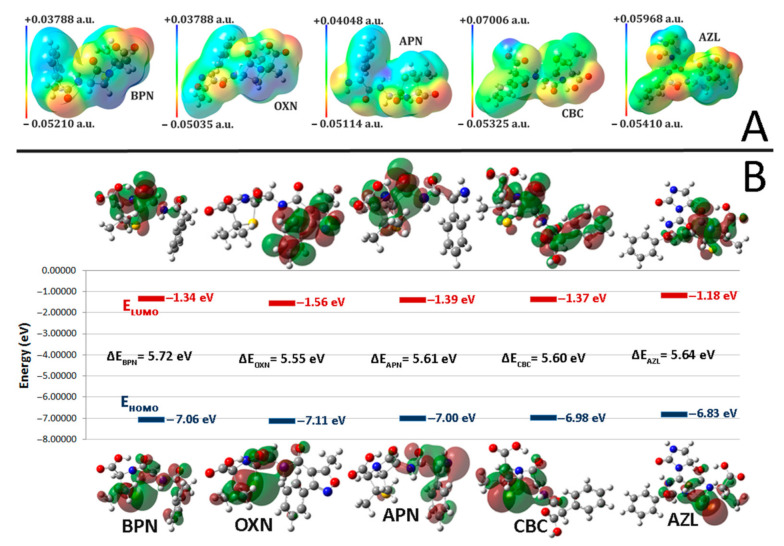
(**A**) Molecular electrostatic potential surfaces (MEPs) for optimized structures of BPN, OXN, APN, CBC, and AZL, respectively. Surface color ranges from the most electropositive areas (blue) to the most electronegative ones (red). The electron density isosurface is 0.02 a.u. (**B**) Frontier molecular orbital (FMO) diagrams for BPN, OXN, APN, CBC, and AZL, calculated at B3LYP/6-311+G(2d,p) level of theory.

MEPs are used as illustrative tools for determining the reactive sites of a ligand that are responsive to surrounding nuclei or electrons in order to form hydrogen bonds or for electrophilic and nucleophilic attacks. We obtained MEPs of BPN, OXN, APN, CBC, and AZL (Figure 2A) after optimizing their geometry by using DFT methods at the B3LYP/6-311+G(2d,p) level of theory, with an electron density isosurface of 0.02 a.u. The MEP values were calculated as previously described by Polizer and Murray [39]. As seen in Figure 2A, the colored illustration of a MEP varies from its most electronegative areas, pictured in red, to its most electropositive areas, colored blue. The two extremes—the hydrophilic areas—meet in neutral zones (green areas), also known as the hydrophobic regions. The transition from the most negative to the most positive areas is more gradual, from red to orange, yellow, green, cyan, and blue, as seen in Figure 2A, in the color scale of each MEP.

It is clear that for all compounds, the most electronegative sites are concentrated on the O atoms in carbonyl groups, whereas the most electropositive sites are localized on the N atoms. In addition, another electronegative area in OXN’s MEP is located on O and N atoms in the isoxazole ring; for APN, electronegative sites are located on the O in the second carboxyl’s hydroxyl as well as on the amide’s N atom. The negative regions scored values between −0.05410 a.u. (AZL) and −0.05035 a.u. (OXN); these regions are the most likely to be targeted for electrophilic attacks or to be involved in forming hydrogen bonds. The electropositive areas’ location varies between the five antibiotics: On BPN and OXN, the most electropositive sites are located on C atoms (from CH groups) in 6-APA, followed by N in NH, and C atoms in the benzene ring and methyl groups; the most electropositive area on APN is located on the N atom in NH, followed by the C of the CH group in the β-lactam ring; the most electropositive area on CBC’s MEP is located on hydroxyl’s O atom, scoring the maximum value in the class (+0.07007 a.u.); lastly, the most electropositive area for the AZL molecule, scoring the second most electropositive value (+0.05968 a.u.), is located on the N atom in NH from the imidazolidine ring. These sites are most likely to be involved in nucleophilic attacks or to form hydrogen bonds. The values for all three other penicillins are equal to or smaller than +0.04 a.u., with the difference between the maximum (CBC) and minimum (BPN) values of the electropositive areas being more than 0.03 a.u.

### 2.3. Frontier Molecular Orbitals (FMO)

Frontier molecular orbitals (FMOs), comprising the highest occupied molecular orbital (HOMO) and the lowest unoccupied molecular orbital (LUMO), are key indicators for providing information on a molecule’s chemical reactivity and stability. When two molecules interact with each other, the HOMO of one molecule will interact with the LUMO of the second. Therefore, identifying the regions of the electron density and the energy of the frontier orbitals in a molecule is essential when one wants to describe intermolecular interactions. Figure 2B shows the distribution of the charges in the FMOs with the negative (red) and positive (green) values of the orbitals, as well as their energy levels and the (HOMO-LUMO) band gap (HLG). The HOMO energy of a molecule indicates its electron-donating capacity. Electrons localized in the HOMO position are the most inclined to engage in reactions, whereas the LUMO energy indicates the electron-accepting capacity of a molecule. The regions of the electron density in LUMO indicate the most electrophilic side of a molecule.

The electron density of HOMO is localized on the same chemical groups in BPN, OXN, CBC, and AZL, primarily on the S atom, followed by C atoms in methyl groups and C atom bonded to them, as well as on the C atoms in the *β-lactam* ring and on the amide and carbonyl groups. HOMO spreads on the benzene ring in BPN as well, on the O atom in the *β-lactam* ring in CBC, and on the second amide group in AZL. The APN is an exception. The electron density of HOMO spreads mainly on the benzene ring, the amino group, and the C atoms bound to it, the same amide and carbonyl groups, and the S atom.

The electron density for LUMO in BPN, APN, CBC, and AZL molecules spreads mainly over the *β-lactam* and *thiazolidine* rings, amide and carbonyl groups, and the carbonyl group in the carboxyl. For CBC, LUMO is localized on the benzene ring and on its specific carboxyl group, while the LUMO in OXN is localized only on the benzene and isoxazole rings.

Based on Koopmans’s theorem [40], which states that the first ionization energy of a molecule is equal to the negative value of the HOMO’s energy, several parameters have been described over time for better characterization of the reactivity of chemical compounds, such as ionization potential (I), electron affinity (A), (HOMO-LUMO) band gap (HLG), global hardness (η) and softness (σ) [41], electronegativity (χ) [42] and its reverse, the chemical potential (μ), and the global electrophilicity index (ω) [43,44]. We calculated all these parameters for BPN, OXN, APN, CBC, and AZL. The results are listed in Table 3.

The charge transfer interactions between a molecule and its surroundings are described by HLG. Its value represents the quantity of energy required to remove an electron from HOMO. In general, the smaller the value of HLG, the faster the reaction will occur. HLG is the most important stability descriptor of a molecule. More reactive molecules have a smaller HLG value, which helps them to donate their electrons to an acceptor more easily. As shown in Table 3, the difference between the lowest and the largest band gap is not significant (slightly over 0.15 eV). With the lowest HGL of the five compounds, OXN presents the highest chemical reactivity, followed by CBC, APN, and AZL, while BPN presents the highest stability. On the other hand, HLG values of 5.5 eV and above indicate that all five compounds are stable. The electrophilicity index, another measure of a system’s ability to accept electrons, increases from AZL to OXN, indicating that AZL is the most stable of the five, while OXN is the most willing to accept electrons from the environment. A good nucleophile is characterized by low values of μ and ω, thus making AZL the most likely compound to be involved in a nucleophilic attack.

Thus, the ligands with the lowest value for HLG and the lowest global hardness as well as the highest softness are the most likely to present bactericidal properties. From the five penicillins, OXN scores the above-mentioned values. On the other hand, the ligand showing the least electronegativity and electrophilicity while presenting the highest value for the chemical potential is the most likely to be a good bactericide; in this case, this is AZL.

In conclusion, the ligands with less electronegativity (AZL), less hardness (OXN), less global electrophilicity (AZL), a small value of HLG (OXN), more softness (OXN), and more chemical potential (OXN) are more reactive and have better bactericidal properties.

### 2.4. Molecular Docking

Autodock Vina [45] is an automated routine employed to predict ligand–receptor interactions. It combines an efficient scan for all available degrees of freedom, such as torsion angles of the system, with a rapid grid-based energy evaluation. By using a search algorithm and an empirical free energy scoring function, the code refines the multitude of possible docking geometries to the most probable one—the one with the lowest binding energy between the receptor and the ligand. This algorithm is non-deterministic, which causes every search to generate random results. Thus, for each ligand–receptor pair of the twenty systems (five ligands × four receptors), we set the number of binding modes to be generated to 10 per code run, keeping the default = 8 value of the exhaustiveness. We run the code one hundred times, resulting in 1000 docking conformations. The distribution of the conformers with the lowest values of the binding energy for each of the hundred runs for all twenty ligand-receptor systems is pictured in Figure S2. Following the same steps described in our previous work [46], we consider this number to be large enough to allow us to characterize the antibiotics-PBPs systems from a combination of the predicted binding energy and geometrical specificity point of view.

We predicted the binding affinity of BPN, OXN, APN, CBC, and AZL penicillins to the PBPs from *A. hydrophila*, *M. morganii*, *B. cereus*, and *E. faecalis* by molecular docking. The smallest binding energy, as well as the average value scored by each ligand, are listed in Table 4. Hydrogen bonds (HB) are described by listing the specific atoms and residues involved, as well as the type of bond and their lengths.

**Figure 3:**
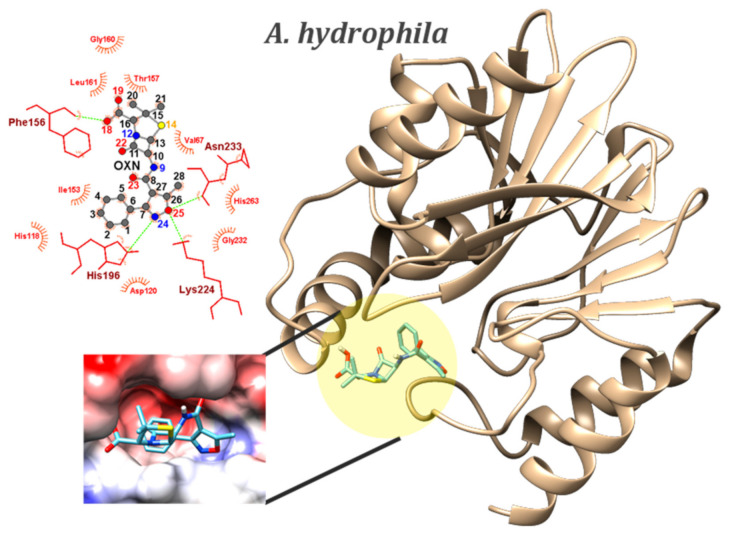
Position of the binding sites of CphAs from GN *A. hydrophila* (PDB id: 1x8i) in complex with OXN, which scored the lowest binding energy (−8.8 kcal/mol). Zoomed-in pictures show the Coulombic electrostatic surface of the binding sites and OXN–residues interactions, with emphasis on the hydrogen bonds (dashed green lines).

The binding site of *A. hydrophila* is localized at the surface of the CphA and has a crevice-like shape (see Figure 3). Both electropositive and electronegative areas can be localized on its surface. The best binding energy for this receptor was scored by the classic OXN molecule, with an average of −8.70 ± 0.02 kcal/mol and a minimum registered at −8.8 kcal/mol, scored by 4 conformers out of 100 code runs. Biapenem, the co-crystalized ligand, displayed binding energy of −9.4 kcal/mol, smaller than OXN by 0.6 kcal/mol. Biapenem is a *β-lactam* as well, but it belongs to the carbapenem class, which is known to have a broader spectrum of activity than most penicillins and cephalosporins, particularly against Gram-negative bacteria [47]. Three of the O atoms in biapenem are involved in HB with HIS196, LYS224, and ASN233 residues. The OXN molecule is involved in HB with the same residues as biapenem—HIS196, LYS224, and ASN233, with N24 and O25 atoms as acceptors. According to Jeffrey’s rule [48], these hydrogen bonds are of moderate strength. O18 from the hydroxyl group forms a weak HB, this time as a donor, with PHE156 and THR157. The area on the MEP of the isoxazole ring around the two atoms is negatively charged (see Figure 2A) and its position in the binding site of *A. hydrophila* is directed towards an electropositive area (as seen in Figure 3). All three atoms involved in HBs (N24, O25 and O18) were previously identified as being the most likely to be involved in electrophilic attacks. With a difference of 0.5 kcal/mol for the minimum binding energy scored, CBC and AZL come in as the second most effective against *A. hydrophila*. Generally, the same residues of the receptor are involved in HB as for CBC, O atoms in carboxyl and carbonyl groups, while for AZL, there are those from the carboxyl group and its particular two carbonyl groups. In both cases, all atoms’ MEP values were the most negative on all these atoms, thus an electrophilic attack on these atoms, or being involved in HB, is the most probable outcome.

**Figure 4:**
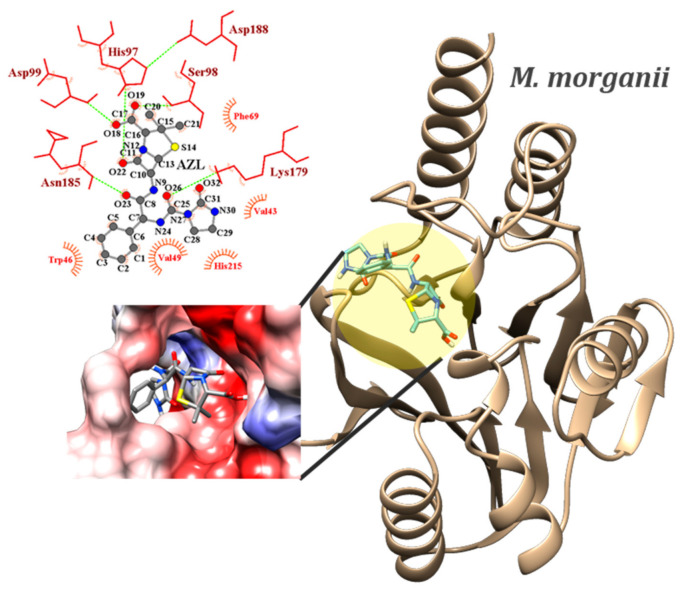
Position of the binding sites of PBPs from GN *M. morganii* (PDB id: 6l3s) in complex with the AZL conformer, which scored the lowest binding energy (−8.0 kcal/mol). Magnified pictures show the Coulombic electrostatic surface of the binding sites and AZL–residues interactions, with emphasis on the hydrogen bonds (dashed green lines).

The active site of *M. morganii* is slightly retracted within the protein’s structure, giving it a cave-like appearance. The active site’s shape might imply a better specificity for ligand geometry. Two of the explored compounds in this study, OXN and AZL, scored the minimum binding energy of −8.0 kcal/mol, with their average values being close (−7.81 ± 0.06 kcal/mol vs. −7.86 ± 0.08 kcal/mol). However, AZL has a standard deviation (sd) error of just over 1%. A comparison of our results with those from a co-crystalized ligand was not possible for the case of *M. morganii* since its active site’s position is indicated just by a Zn atom, and no information about inhibitors’ binding modes to PBP was found in the literature. OXN is involved in a moderate HB with SER98 and a weak HB with ASP99 through O19 and O18, making them the most likely atoms from OXN to be involved in electrophilic attacks. The carboxyl group is oriented toward the binding site electropositive (blue) area (see Figure 4). Through O18, O19, O23, O26, and O32, the most likely atoms of AZL to be involved in electrophilic attacks, the molecule forms moderate HB with ASP99, SER98, ASN185, and LYS179, respectively. The N30 atom in the imidazolidine ring acts as a donor to GLY182 in a weak HB. CBC scored a 0.4 kcal/mol higher binding energy. Its O atoms interact with the same residues.

**Figure 5:**
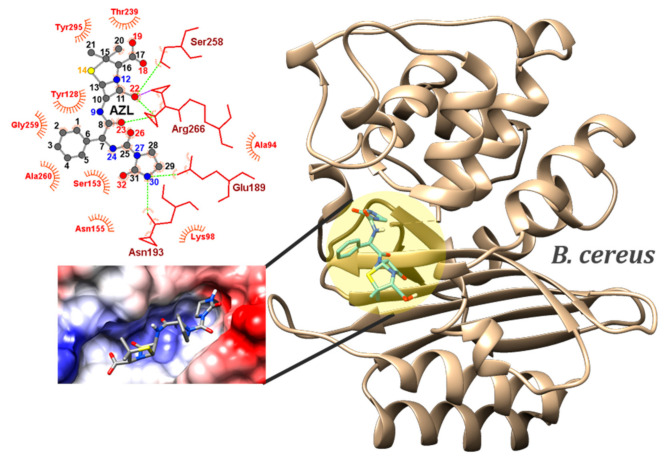
Position of the binding sites of PBPs from GP *B. cereus* (PDB id: 6w33) in complex with AZL conformer, which scored the lowest binding energy (−8.6 kcal/mol). Magnified pictures show the Coulombic electrostatic surface of the binding sites and AZL–residues interactions (left), with emphasis on the hydrogen bonds (dashed green lines).

In the competition for the best bactericide against two pathogens in the Gram-positive class, AZL has notably detached itself from the rest of the compounds, scoring minimum binding energy of −8.6 kcal/mol against *B. cereus* and *E. faecalis*. Clavulanate (clavulanic acid), the co-crystalized ligand scored a binding energy of −4.5 kcal/mol. It is known that clavulanate has little or no bactericidal activity when used alone, and that is most effective when combined with another *β-lactam* [49]. It acts as a suicide inhibitor, binding to a serine residue [50]. Our results indicated SER258 in *B. cereus*’s PBP to act as an acceptor in three HB with the co-crystalized ligand. AZL is involved in multiple HB with ASN155, ASN193, SER258, ARG266 through O18, O22, O23, O26, and O32 (Figure 5). Except for ARG266…O22, which is moderate in strength, the rest of the HBs are weak. The N30 and O18 act as donors in two moderate HBs with ASN193 and THR239. The most electropositive spot on AZL’s EPS, the imidazole ring, has its N30H oriented towards an electronegative spot in the active site, confirming N30’s reactivity potential as being the most likely atom of AZL to be involved in a nucleophilic attack. The other penicillins’ average binding energies are approximately −8.0 kcal/mol (CBC and OXN), or significantly less (APN, BPN).

**Figure 6:**
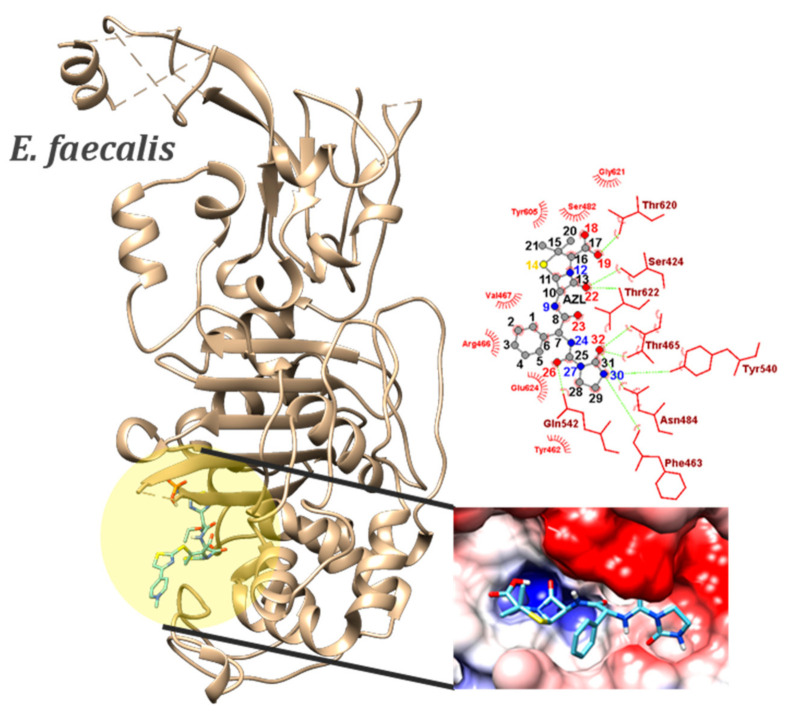
Position of the binding sites of PBPs from GP *E. faecalis* (PDB id: 6mkh) in complex with AZL conformer, which scored the lowest binding energy (−8.8 kcal/mol). Magnified pictures show the Coulombic electrostatic surface of the binding sites and AZL–residues interactions (right), with emphasis on the hydrogen bonds (dashed green lines).

The average binding energy of AZL to *E. faecali*’s PBP was −8.44 ± 0.18 kcal/mol, while all the other compounds had a lower affinity. SER424…O22, THR465…O32, SER482…N12, ASN484…O23, ASN484…O32, and GLN542…O26, as well as two weak HBs—THR620…O18 and THR622…O22—are formed between PBP’s residues and atoms in imidazolidine and thiazolidine rings, carbonyl, carboxyl, and amino groups (Figure 6). In three HBs, O18, N24, and N30 act as donors as well. When docked to *E. faecali*’s PBP, AZL exhibits the highest specificity. From 100 docking experiments, 46 of the best conformers in each run scored a binding energy of −8.6 kcal/mol, whereas imipenem, the co-crystalized ligand, scored only −5.5 kcal/mol. In particular, imipenem is known to present bactericidal activity against both Gram-positive and Gram-negative bacteria, especially for *Enterococcus* species and *Pseudomonas aeruginosa* [51]. It is involved in four HB, two with the donors SER482 and ASN484 and two with the acceptors THR620 and SER424; all four residues interact with AZL as well.

The key outcomes of the docking studies show that, in terms of chemical reactivity, both a newer-generation antibiotic (AZL) and an older-generation antibiotic (OXN) are effective against various penicillin-binding proteins responsible for Gram-positive and Gram-negative bacterial infections. The same two penicillins—OXN and AZL—were identified as being the most reactive ligands from both the MEPs description and the Frontier Molecular Orbital studies.

### 2.5. Disk Diffusion Tests

The bactericidal activities of the selected antibiotics on both GN and GP groups are summarized in Table 5 as inhibition zones expressed in mm, or R as *resistant* if there is no inhibitory action. The susceptibility to the selected antibiotics varies both from the GN to the GP group and also within the groups. The *A. hydrophila* PI-88 strain is sensitive to APN, CBC, tetracycline (TRC), and AZL, being resistant to BPN and OXN, while the strain PAI-45 is resistant to APN, CBC, BPN, and OXN, and sensitive to TRC and AZL (Figure S3). *M. morganii* presents resistance to all six tested antibiotics (Figure S4).

For the GP strains, *B. cereus* proved to be resistant to all antibiotics tested (Figure S5), while *Enterococcus* species presented resistance only to OXN. Their sensitivity increases from CBC, BPN, to AZL. APN presents the greatest bactericidal effect on *E. lactis* CE-13 and *E. durans* CI-28. The sensitivity to TRC of the pathogens from the GP group was not tested (Figure S6).

In general, in the GN class, OXN scored the lowest binding energies to both receptors, whereas the disk diffusion tests indicated that all tested pathogens exhibited resistivity to OXN. While they were ranked as the most second reactive antibiotics in the docking study, disk diffusion tests indicated CBC and AZL were the most efficient against GN *A. hydrophila*. However, in practice, the species shows resistance to OXN, despite OXN scoring the lowest binding energy. Experimental validation of the docking results for the GN *M. morganii* was not possible due to the pathogen’s antibiotic resistance. The lowest binding energy scored for *M. morganii’s* PBP was −8.0 kcal/mol, being, at the same time, the weakest binding energy of a conformer in the whole study. This might be an indication of weak interaction, which might correlate the docking results with reality.

In the GP class, *B. cereus* showed the same predicament as *M. morganii* since the grown strain showed resistance to all tested compounds. As compared to docking results, where the fifth-generation penicillin AZL scored the lowest binding energy (−8.6 kcal/mol), the disk diffusion test results for *Enterococcus* strains indicated that APN performed best, followed by AZL and CBC.

In the end, we can state that the strongest binding energy of a conformer resulting from a molecular docking study can provide some insight into the chemical structure properties for antibiotics’ design and formulation, but this does not always imply that, in reality, the compound is the most efficient option for bactericidal action against a pathogen. The disparity between the obtained theoretical and experimental results is also due to the discrepancy between the selected PBPs used in the docking studies and the pathogenic strains that were available in our lab for disk diffusion tests. On the other hand, OXN might have been good bactericidal penicillin at some point (the theoretical results indicated OXN as being one of the most reactive compounds in our study), but the overuse and misuse of antibiotics led to OXN resistivity for both GN and GP bacteria, even the common ones.

### 2.6. Bacterial Resistogram Based on SERS and PCA

We have monitored the SERS bacterial response of *A. hydrophila* species in three scenarios: A *control case* (C)—pathogen culture in a liquid medium, without antibiotic treatment; a *sensitivity case* (S)—a sensitive response to the antibiotic treatment; and a *resistance case* (R)—pathogen showing resistance to antibiotic treatment. The three scenarios were compared, taking into account the bacterial SERS profiles recorded at the single-cell level, using a monitoring protocol developed by our research group, described in previous reports [25,26,52]. The results are not collected in *real time,* but they still enable clinically relevant insight into the antibiotic’s action in a turn-around time (TAT) of a working shift (8–12 h).

**Figure 7:**
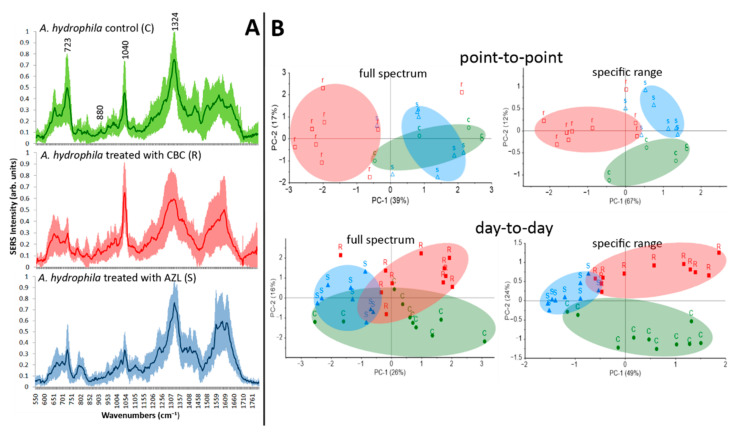
(**A**) SERS profiles and average (darker color) spectrum of *A. hydrophila* for the control case (C) (without antibiotic treatment) and treated with antibiotic when the pathogen shows resistance (R) or sensitivity (S). (**B**) 2D scores plots for PC-1 and PC-2 and the visibly formed C (green), R (red), and S (blue) clusters. PCA was performed on the full spectral range (left plots) and on a specific range (right plots). Samples were recorded on the same day (top row) or on separate days (bottom row).

Figure 7A shows the characteristic SERS signatures for the C, S, and R situations. SERS bands specific to adenine (723 cm^−1^ and 1324 cm^−1^), generally considered pathogen viability marker bands [22,23], are present for C and R scenarios, with significantly increased intensity compared to S. In other words, efficient antibiotic therapy affects the pathogen’s SERS profile when working primarily on the cell-wall synthesis, causing the adenine derivatives present in its composition to be detected with lower intensities. The ratios between the intensities I_723_ and I_1324_ of the SERS bands found in the specific microbial fingerprint are considered quantifiable parameters for SERS-based AST [53].

The next step was to combine SERS spectroscopy and PCA to create a classification model able to discriminate between *A. hydrophila* samples treated with an antibiotic for which they show resistance (CBC) and sensitivity (AZL) in order to obtain a *SERS-based resistogram*. The pretreatment and data analysis using PCA have been described in several of our previous works [54,55,56].

First and foremost, we need to demonstrate the point-to-point reproducibility of our data. To do so, we performed a PCA on a database of spectra recorded on the same day. When running the PCA, the entire spectral range (550–1800 cm^−1^) was taken into consideration. The first two PCs obtained added up to the total variance of 56%. R samples were grouped into a wide cluster, while S and C clusters merged (Figure 7B). All the analyzed SERS spectra are plotted in Figure 7A, together with the average spectrum of each case, where narrow bands at 880 cm^−1^ and 1040 cm^−1^ and the wide band spreading over 600–750 cm^−1^ with variable intensities from R to S cases can be observed. On the other hand, the intensities are reproducible for spectra belonging to the same group. 1040 cm^−1^ is the marker band with the biggest contribution for all first three PCs (see loadings plots in Figure S7), together with the two marker bands specific to adenine. Taking this into account, we performed a second PCA, where only the wavenumbers describing the aforementioned bands were chosen as variables. Even though some S and R satellite samples drifted from their group, C and S clusters separated visibly from the mix. In this case, the first three PCs added up to an improved total variance of 84%, meaning that an unknown sample could be assigned to the group to which it truly belongs with an 84% success rate.

Secondly, we performed a PCA on a database containing *A. hydrophila* treated with antibiotics and SERS fingerprints recorded on separate days to demonstrate the day-to-day reproducibility. When the whole spectral range was considered as variables, the data were grouped into two clusters, R and S, while the C samples spread in a wider area, also interfering with the other two clusters. The model has a total variance of 52% for the first three PCs. The total variance improved to 77% for a second PCA when only the wavenumbers in the previously defined specific spectral range were used as variables. The three clusters were clearly separated from one another as well.

## 3. Materials and Methods

### 3.1. Sample Preparation

#### 3.1.1. Chemicals

Penicillin G sodium salt (CAS 69-57-8), ampicillin (CAS 69-53-4), and carbenicillin disodium salt (CAS 4800-94-6) were purchased from Alfa Aesar GmbH & Co KG (Thermo Fisher Scientific, Kandel, Germany). Oxacillin sodium salt (CAS 1173-88-2) and azlocillin sodium salt (CAS 37091-65-9) were purchased from Sigma Aldrich Handels Gmbh (Wien, Austria) at reagent grade.

#### 3.1.2. Bacterial Strains

*Aeromonas hydrophila* PAI-45 and PI-88, and *Morganella morganii* PI-81 were chosen as the GN microbial strains, while *Bacillus cereus* ESN-09, *Enterococcus lactis* CE-13, and *Enterococcus durans* were the GP microbial strains in order to test their susceptibility to the five antibiotics.

#### 3.1.3. Disk Diffusion Tests

Each strain was first cultivated in an LB medium as a unique colony, with the inocula concentration being further adjusted to 1.5 × 10^8^ CFU/mL (0.5 McFarland). Then, 0.5 mL of the resulting suspension of each strain was transferred to Petri dishes containing the Muller–Hinton Agar Medium. Further, 6 mm sterile disks containing antibiotics were placed in the Petri dishes and set for incubation for 24 h at 35–37 °C. After the incubation, the circular growth zones were measured with a digital caliper. Antibiotics used on the white disks were 10 μg of benzylpenicillin, 5 μg of oxacillin, 10 μg of ampicillin, 100 μg of carbenicillin, 30 μg of azlocillin, and 30 μg of tetracycline per disk.

### 3.2. Vibrational Analysis

#### 3.2.1. FT-Raman Analysis

FT-Raman spectra were recorded using a Raman Bruker FRA 106/S accessory attached to an FT-IR Equinox 55 spectrometer (Bruker Optik GmbH, Ettlingen, Germany). The excitation source was a 1064 nm laser of 250 mW power. The spectrometer was equipped with a Ge detector, cooled with liquid nitrogen. The final FT-Raman spectrum contained 120 scans with a resolution of 2 cm^−1^.

#### 3.2.2. In Situ Silver Nanoparticles (AgNPs) Synthesis

The SERS active colloidal system was synthesized in situ, as reported by Zhou et al. [26], in the presence of bacteria. For a final volume of 1 mL, 10 μL of bacterial biomass was immersed in the 100 μL silver nitrate solution (10^−3^ M) and then 900 μL of the reducing agent (hydroxylamine hydrochloride, 10^−3^ M, and NaOH 1% mixture). This procedure was used to generate in situ AgNPs at the bacterial cell wall structure. After approximately 3 min of silver nanoparticles/biomass interaction, SERS measurements were initiated on 3 μL sample spots immobilized onto microscopic slides.

#### 3.2.3. SERS Spectra Measurements

SERS profiles were recorded using the NTEGRA Spectra platform (NT-MDT BV, Apeldoorn, The Netherlands), equipped with a Raman confocal SOLAR TII spectrometer and an Olympus IX71 microscope, by using the 100× objective (NA 0.7) and the 532 nm laser line (<0.1 cm^−1^ spectral resolution) with a total power of 100 mW. The effective power used on the sample was determined as 11 mW. Thirty accumulations with a 3s acquisition time were recorded at the single-cell level.

### 3.3. Computational Details

#### 3.3.1. Input Files for DFT Calculations

Before proceeding on the computational pathway, two-dimensional (2D) relaxed potential energy surfaces (PES) were generated aiming to identify the global minimum for each molecule. The global minimum was found following the next steps: Starting from the 2-azetidinone group (β-lactam ring), the first two dihedral angles, corresponding to the first two bonds of the R chain (one of them being the amino group at position 6), were each modified with a step of 15°. During the geometry optimization process, these dihedral angles were kept frozen, while the rest of the molecule was relaxed. Finally, the 2D scan counted 576 steps (24 × 24 steps). Each molecule received three such scans. The second scan was performed by freezing the second and third dihedral angles. The initial geometry used for this second scan was the resulting global minimum in the first scan. The steps followed were the same for the third scan: The global minimum determined in the second scan was used as an input geometry, while the third and fourth dihedral angles were kept frozen.

DFT methods were used for all computations, which were carried out with the Gaussian 09, Revision E.01 software package [57]. Both geometry optimization and frequency calculations were performed using B3LYP hybrid exchange-correlation function [58,59,60,61] coupled to the 6-31G(d) [62,63] (for anharmonic approximation) and 6-311+G(2d,p) basis sets (for harmonic approximation). Wavefunction convergence and geometry optimization were met using very tight criteria. The ultrafine grid option was chosen for the integration of the electronic density. According to the results obtained from frequency calculations, all of the optimized geometries correspond to true minima on the potential energy surface (no imaginary frequency was obtained). The matching scaling factor of the used level of theory, 0.967 [64], was used for harmonic frequencies. We considered both the relative intensities and peak positions of the calculated Raman spectra when assigning the normal modes at harmonic and anharmonic levels.

#### 3.3.2. Selecting the Ligands and Receptors

Before proceeding to molecular docking calculations, we started by preparing the input files. For the ligands, we used the previously optimized geometries of BPN, OXN, APN, AZL, and CBC. Next, we chose four PBPs belonging to four different bacteria as receptors, two for each of the GN and GP classes. We selected the crystal structures of the receptors from the Protein Data Bank (PDB) as follows: Chain A of the zinc carbapenemase CphA from GN *A. hydrophila* (PDB id: 1x8i) [65], with a resolution of 1.90 Å, chain A of metallo-beta-lactamase IMP-27 from GN *M. morganii* (PDB id: 6l3s) [66], with a resolution of 1.70 Å, chain A of class A beta-lactamase from GP *B. cereus* (PDB id: 6w33) [67], with a resolution 1.85 Å, and chain A of PBP4 from GP *E. faecalis* (PDB id: 6mkh) [68], with a resolution of 2.62 Å. We removed all water molecules and all ligands or cofactors, if any, from the original files before building the ligand–receptor systems. This step was performed in Molegro Molecular Viewer 2.5 (Molexus Ivs, a CLC biocompany, Odder, Denmark).

#### 3.3.3. Building the Ligand-Receptor Systems

We built the ligand–receptor systems in Autodock Tools 4 [69] by combining each of the five antibiotics with each of the four receptors, resulting in a total of twenty such systems. We added only polar hydrogens to the receptors’ structures and set them to be kept rigid while running the docking algorithm against the ligands, for which we allowed maximum flexibility. For the latter, we set the number of torsion angles to six for BPN and OXN, seven for APN, nine for AZL, and eight for CBC.

#### 3.3.4. Setting the Search Box

In order to be sure that the studied ligands bound exactly to the active site of the considered receptors, we first proceeded to the confirmation of the binding site indicated in the crystal complex by the original inhibitor. As a result, we removed the original inhibitor from the crystallized ligand–receptor complexes and performed the docking to their corresponding receptors by using a search box the size of the whole receptor. The re-docked complex was overlaid onto the crystallized form and confirmed that the position of the binding site (Figure S1). Further, for the actual docking of the twenty considered systems in this study, we chose the search boxes to enclose the binding sites of the original ligands in the crystal structures of the macromolecules. The search box for *A. hydrophila* is centered to (19, 47, 67) sized (24, 24, 24); *M. morganii* PBP is centered to (8, −1, 31) sized (25, 25, 25); *B. cereus* PBP is centered to (4, −4, 7) sized (25, 25, 25); and *E. faecalis* PBP is centered to (36, 0, 8) sized (28, 28, 28). The grid spacing was set to 1 Å for all grid boxes.

#### 3.3.5. PCA Analysis

The initial database used for multivariate analysis contains SERS spectra for the Gram-negative *A. hydrophila* with and without antibiotic treatment. Baseline subtraction and normalization relative to the highest peak were performed on the original SERS measurement. These pretreatments were performed using SpectraGryph software (2001–2017). For the multivariate analysis, seven principal components (PCs) were calculated using The Unscrambler X 10.4 (developed by CAMO Software AS., Oslo, Norway).

## 4. Conclusions

With the aid of DFT frequency calculations, a complete assignment of the Raman spectra of five penicillins—one for each generation—was described. We aimed to reveal the (dis)similarities in their chemical structure by comparing their Raman responses. As a result, we draw the conclusion that penicillins come with a specific Raman response, with the band at 1002–1004 cm^−1^ being the most intense in all spectra. However, even if subtle, each antibiotic presented specific Raman marker bands in its spectrum, which were absent in all others. The enhanced intensity of 1606 cm^−1^ (C6C7 stretching), 1444 cm^−1^ (C28H_3_ bending), and 1471 cm^−1^ (C7=N24 and C26C27 stretching) makes OXN’s Raman spectrum stand out the most. More visible changes may also be seen in the case of APN at 780 cm^−1^ (NH bending) and 830 cm^−1^ (NH_2_ twisting).

Based on the analysis of the frontier molecular orbitals (FMOs) and the molecular electrostatic potential surfaces (MEPs), it was possible to determine the chemical reactivity and correlated efficiency towards specific GN and GP pathogenic strains against OXN, CBC, AZL, and two other classical penicillins. When the global reactivity descriptors were taken into account, OXN has the strongest bactericidal activity, having the highest values for I and ω, and the lowest value for HLG. Regarding its HLG value, CBC was ranked as the second-best bactericide compound.

The key outcomes of the docking studies demonstrated that both a newer-generation antibiotic (AZL) and an older-generation antibiotic (OXN) are effective against various penicillin-binding proteins responsible for Gram-positive and Gram-negative bacterial infections. OXN scored the strongest binding energies to the CphA in GN *A. hydrophila* (pdb id: 1x8i; −8.70 ± 0.02 kcal/mol) and GN *M. morganii* (pdb id: 6l3s; −7.86 ± 0.08 kcal/mol), whereas AZL scored the strongest binding energies to the PBPs in GP pathogens *B. cereus* (pdb id: 6w33; −8.32 ± 0.12 kcal/mol) and *E. faecalis* (pdb id: 1x8i; −8.44 ± 0.18 kcal/mol). Their bactericidal activity was tested and confirmed on a couple of both Gram-positive and Gram-negative species using the disk diffusion method. Aside from the novel reported data, the methodology of our study, starting with the spectroscopic and reactivity characterization of the active compounds and continuing with a molecular docking study validated by disk diffusion testing, can be extended to any class of beta-lactams for a broader understanding of the mechanism of action of such bactericides. One such example is the cephalosporin class, from which a selection of compounds is to be the subject of future work. Additionally, a SERS–PCA-based *resistogram* of *A. hydrophila* is proposed as a clinically relevant insight resulting from the synergistic cheminformatic and vibrational study on CBC and AZL.

## Figures and Tables

**Table 1:** Chemical structure of penicillins, BPN, OXN, APN, CBC, and AZL together with the pathogens presenting specific sensitivity or resistance to each of them.

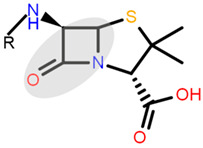 Chemical structure of 6-APA and the position of the side chain R in penicillin’s structure; *β-lactam ring* is marked in grey.			
**COMPOUND** **FAMILY** **(GENERATION)**	**CHEMICAL STRUCTURE**	**SENSITIVITY**	**RESISTANCE**
**BPN** β-lactamase sensitive (1st generation)	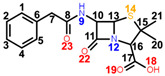 **C_16_H_18_N_2_O_4_S**	**narrow spectrum** Gram-positive (GP) bacteria *Neisseria gonorrhoeae* *Leptospira weilii*	*Escherichia coli* *Salmonella typhi*
**OXN** β-lactamase resistant (2nd generation)	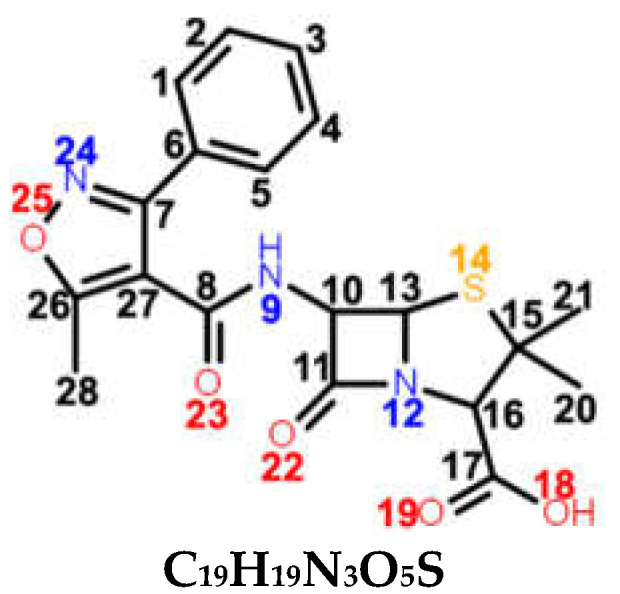 **C_19_H_19_N_3_O_5_S**	**narrow spectrum** Gram-positive bacteria PRSA	ORSA, MRSA
**APN** aminopenicillin (3rd generation)	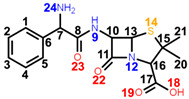 **C_16_H_19_N_3_O_4_S**	**broad spectrum** *Streptococcus* spp. *Enterococcus* spp. *Neisseria meningitidis* *Haemophilus influenzae*	MRSA, PRSA *Enterobacteriaceae* spp. *Pseudomonas* spp.
**CBC** carboxypenicillin (4th generation)	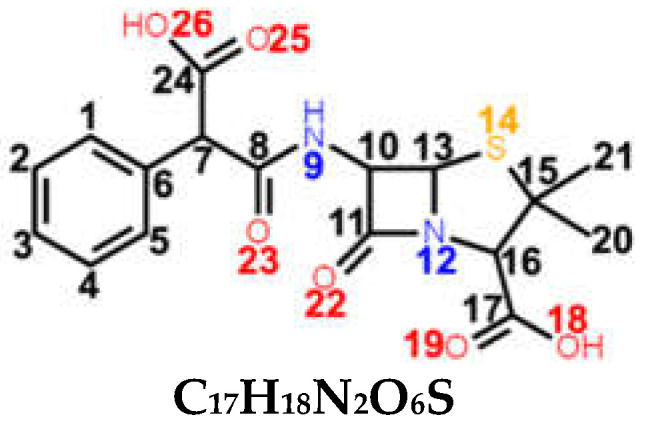 **C_17_H_18_N_2_O_6_S**	**broad spectrum** *Escherichia coli* *Proteus mirabilis* *Pseudomonas aeruginosa*	
**AZL** ureidopenicillin (5th generation)	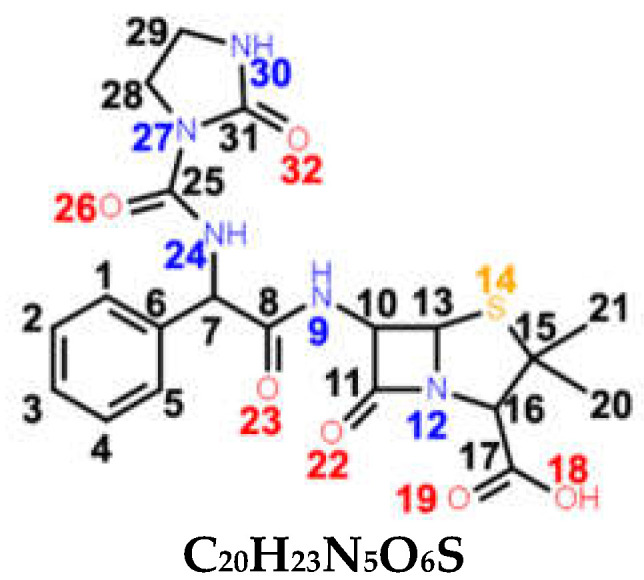 **C_20_H_23_N_5_O_6_S**	**broad spectrum** *Escherichia coli* *Haemophilus* spp. *Pseudomonas aeruginosa*	

**Table 2:** Experimental (1064 nm) Raman marker bands of penicillins (common to all five penicillins) and specific Raman bands for BPN, OXN, APN, CBC, and AZL molecules classified by chemical groups.

	BPN		OXN		APN		CBC		AZL		
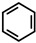 benzene ring	621 839 983 1002 1029 1582; 1600		629 845 982 1002 1026 1578; 1606		615 830 993 1003 1027 1585; 1602		617 834 957 1004 1032 1582; 1600		617 - 988 1003 1030 1585; 1602		**common bands**
CH_3_ methyl group	231 274 292	960 1436 1452; 1468	250 279 296	- 1433 1459	240 271 291	951; 961 1435 1456	246 280 -	948 1436 1457	241 - 294	948; 958 1437 1458	
COOH carboxyl group	360 - 807 -	873 + 895 919 1245	368 522 - -	894; 910 920 -	360 522 802 847	873; 910 - 1249	361 525 803 847	876;891 920 -	360 - - 850	870;899 - -	
NH amide group	661 1178		656 -		670 1178		666 -		661 1175		
C=O carbonyl group	402 1638		406 1649		409 1693		405 1666		409 1660		
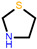 thiazolidine ring	571 602 919 1292		579 616 920 -		- 601 926 -		577 602 920 1297		575 - 913 -		
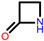 β-lactam ring	945 - 1775		944 - 1758		- 1156 1764		- 1156 1763		- 1158 1775		
CH_2_ methylene group	468 1419		x		x		x		x		**specific bands**
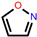 isoxazole ring	x		250 336 492 648 734 793	908 1308 1444 1471 1516 1556	x		x		x		
NH_2_ amino group	x		x		465 830 1119	1186 1512 1638	x		x		
COOH carboxyl group	x		x		x		666 746 1126	1180 1372 1666 1763	x		
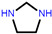 imidazolidine ring	x		x		x		x		465 714 958	1125 1239 1397	
NH amide group	x		x		x		x		641 1532		

**Table 3:** Quantum chemical reactivity descriptors obtained on the optimized geometries of the selected antibiotics by DFT calculations in gas phase at B3LYP/6-311+G(2d,p) level of theory. All values are in eV.

	BPN	OXN	APN	CBC	AZL
**E_HOMO_**	−7.06380	−7.11904	−7.00911	−6.98598	−6.83060
**E_LUMO_**	−1.34288	−1.56275	−1.39540	−1.37880	−1.18804
**I**	7.06380	7.11904	7.00911	6.98598	6.83060
**A**	1.34288	1.56275	1.39540	1.37880	1.18804
**HLG**	5.72092	5.55629	5.61371	5.60718	5.64256
**η**	2.86046	2.77815	2.80686	2.80359	2.82128
**σ**	0.34959	0.35995	0.35627	0.35669	0.35445
**χ**	4.20334	4.34090	4.20226	4.18239	4.00932
**μ**	−4.20334	−4.34090	−4.20226	−4.18239	−4.00932
**ω**	3.08833	3.39136	3.14568	3.11964	2.84882

**Table 4:** Minimum (MIN) and average binding energy (AVE), which includes the standard deviation, for the first conformer in each of the 100 runs of BPN, OXN, APN, CBC, and AZL molecules docked to PBPs (PDB id’s in parenthesis) from *A. hydrophila*, *M. morganii*, *B. cereus*, and *E. faecalis*. The values are expressed in kcal/mol. Hydrogen bonds formed between the most stable conformer of each ligand and surrounding residues in PBPs are also listed.

Class	Pathogen (PDB id)	Ligand	Binding Energy (kcal/mol)	Number of Conformers	HB (D…A)	Type	HB (Å)
Gram-negative	*A. hydrophila* (1x8i)	BIAPENEM (co-crystalized ligand)	−9.4 (MIN)	-	HIS196…O3	NE2…HE2	2.34
					LYS224…O4	NZ…HZ	2.38
					LYS224…O5	NZ…HZ	2.39
					ASN233…O4	N…HN	1.88
		BPN	−8.0 (MIN) −7.83 ± 0.21 (AVE) 2.68% (sd)	29	HIS118…O19	ND1…HD	1.94
					HIS196…O22	NE2…HE2	2.03–2.16
					LYS224…O19	NZ…HZ	2.07–2.16
					LYS224…O18	NZ…HZ	2.28–2.40
					ASN233…O18	N…HN	1.84–1.94
					O18…ASN233	OH…O	2.53–2.66
					O18…THR119	OH…OG1	2.11
		OXN	−8.8 (MIN) −8.70 ± 0.02 (AVE) 0.23% (sd)	4	HIS196…N24	NE2…HE2	2.21–2.23
					LYS224…O25	NZ…HN	2.12–2.18
					ASN233…O25	N…HN	2.00–2.03
					O18…THR157	OH…O	2.48
					O18…PHE156	OH…O	2.25
		APN	−7.7 (MIN) −7.51 ± 0.08 (AVE) 1.07% (sd)	6	HIS118…O19	ND1…HD	1.89–1.93
					LYS224…O22	NZ…HZ	2.22
					ASN233…O22	N…HN	2.13
					N24…PHE156	NH…O	2.40–2.51
					O18…THR119	OH…OG1	2.06
		CBC	−8.3 (MIN) −8.20 ±0.10 (AVE) 1.22% (sd)	38	HIS118…O26	ND1…HD	2.21–2.29
					HIS196…O23	NE2…HE2	2.22–2.29
					LYS224…O22	NZ…HZ	2.34–2.39
					ASN233…O22	N…HN	2.20–2.30
					O26…ASP120	OH…OD1	2.04–2.28
		AZL	−8.3 (MIN) −7.95 ± 0.19 (AVE) 2.39 (sd)	4	GLY160…O18	N…HN	2.57–2.62
					HIS196…O32	NE2…HE2	2.05–2.13
					HIS263…O26	NE2…HE2	2.35–2.47
					O18…THR157	OH…O	2.33–2.46
					O18…PHE156	OH…O	2.07
	*M. morganii* (6l3s)	BPN	−7.4 (MIN) −7.13 ± 0.13 (AVE) 1.82% (sd)	2	SER98…O19	N…HN	2.15–2.17
					ASN185…O23	N…HN	2.28–2.32
					O18…SER98	OH…OG	2.33
		OXN	−8.0 (MIN) −7.81 ± 0.06 (AVE) 0.77% (sd)	4	SER98…O19	N…HN	2.15–2.21
					ASP98…O18	N…HN	2.34–2.35
					O18…SER98	OH…OG	2.08
		APN	−7.3 (MIN) −7.16 ± 0.07 (AVE) 0.98% (sd)	10	SER98…O19	N…HN	2.08–2.23
					ASP99…O18	N…HN	2.23–2.27
					ASN185…O23	N…HN	2.33–2.42
					O18…SER98	OH…OG	2.38–2.59
		CBC	−7.6 (MIN) −7.40 ± 0.16 (AVE) 2.16% (sd)	34	SER98…O19	N…HN	1.90–1.96
					ASP99…O18	N…HN	2.33–2.38
					LYS179…O26	NZ…HZ	2.13–2.26
					ASN185…O25	N…HN	2.06–2.26
					O18…SER98	OH…OG	2.30–2.40
		AZL	−8.0 (MIN) −7.86 ± 0.08 (AVE) 1.02% (sd)	10	SER98…O19	N…HN	2.11–2.21
					ASP99…O18	N…HN	2.17–2.26
					LYS179…O26	NZ…HZ	1.89–1.95
					LYS179…O32	NZ…HZ	2.11–2.18
					ASN185…O23	N…HN	2.19–2.32
					N30…GLY182	NH…O	2.36–2.41
Gram-positive	*B. cereus* (6w33)	CLAVULANATE (co-crystalized ligand)	−4.5 (MIN)	-	N2…SER258 N2…SER258 O1…SER258	NH…O NH…OG O1H…H	2.41 2.13 2.19
		BPN	−7.6 (MIN) −7.26 ± 0.14 (AVE) 1.93% (sd)	2	SER153…N12	OG…HG	2.37–2.38
					SER153…O18	OG…HG	2.16–2.20
					ASN155…O23	ND2…HD	2.16–2.22
					ALA260…O22	N…HN	2.17–2.36
					ARG266…O19	NH1…HH	2.22–2.38
					O18…SER258	OH…OG	2.13
		OXN	−8.5 (MIN) −8.06 ± 0.13 (AVE) 1.61% (sd)	5	ASN155…N24	ND2…HD	2.09–2.24
					ASN193…O25	ND2…HD	2.17–2.22
					SER258…O18	OG…HG	2.20–2.27
					ARG266…O22	NH1…HH	1.99–2.08
					ARG266…O18	NH1…HH	2.38–2.44
					O18…THR239	OH…O	2.36
		APN	−7.7 (MIN) −7.51 ± 0.10 (AVE) 1.33% (sd)	1	SER258…O18	OG…HG	2.03
					ALA260…O23	N…HN	2.11
					ARG266…O22	NH1…HH	1.93
		CBC	−8.2 (MIN) −8.09 ± 0.06 (AVE) 0.74% (sd)	3	LYS98…O23	NZ…HZ	2.71–2.72
					ASN155…O25	ND2…HD	2.15–2.36
					SER258…O18	OG…HG	2.00
					ARG266…O22	NH1…HH	2.00–2.01
					ARG266…O18	NH1…HH	2.31
					ARG266…O18	NH2…HH	2.05
					O26…ASN193	OH…OD1	2.53
					O26…ASN155	OH…DO1	2.41
					O18…SER258	OH…OG	1.99
					O26…GLU189	OH…OE1	2.23
		AZL	−8.6 (MIN) −8.32 ± 0.12 1.44% (sd)	5	ASN155…O26	ND2…HD	2.36–2.39
					ASN155…O32	ND2…HD	2.22–2.26
					ASN193…O32	ND2…HD	2.53–2.57
					SER258…O22	OG…HG	2.34–2.37
					ARG266…O22	NH1…HH	2.34–2.39
					ARG266…O23	NH1…HH	2.54
					ARG266…O18	NH2…HH	2.52–2.56
					ARG266…O22	NH2…HH	1.82
					N30…ASN193	NH…OD1	1.94–1.96
					O18…THR239	OH…O	1.87
	*E. faecalis* (6mkh)	IMIPENEM (co-crystalized ligand)	−5.5 (MIN)	-	SER482…O1 ASN484…O3 N3…THR620 O3…SER424	OG…HG ND2…HD NH…OG1 OH…OG	2.15 2.11 2.36 1.94
		BPN	−7.9 (MIN) −7.61 ± 0.20 (AVE) 1.93% (sd)	4	SER424…O22	OG…HG	1.92–1.98
					THR465…O18	OG1…HG	2.45–2.50
					ASN484…O19	ND2…HD	2.12–2.21
					THR620…O23	OG1…HG	2.18–2.25
		OXN	−7.8 (MIN) −7.70 ± 0.03 (AVE) 0.39% (sd)	6	SER658…O22	OG…HG	1.87–1.90
					O18…SER658	OH…OG	2.20
					O18…GLU635	OH…OE1	1.93
		APN	−7.5 (MIN) −7.27 ± 0.09 (AVE) 1.24% (sd)	1	ASN484…O22	ND2…HD	2.28
					N24…SER482	NH…OG	2.32
					O18…THR465	OH…O	2.21
		CBC	−7.7 (MIN) −7.50 ± 0.10 (AVE) 1.33% (sd)	5	SER424…O22	OG…HG	1.81–2.23
					ASN484…N12	OG…HG	1.99–2.06
					THR620…O25	ND2…HD	2.40–2.46
					THR622…O18	N…HN	2.39–2.59
		AZL	−8.6 (MIN) −8.44 ± 0.18 (AVE) 2.13% (sd)	46	SER424…O22	OG…HG	1.76–2.05
					THR465…O32	N…HN	1.91–2.00
					SER482…N12	OG…HG	2.06–2.22
					ASN484…O23	ND2…HD	1.92–2.04
					ASN484…O32	ND2…HD	1.83–1.92
					GLN542…O26	NE2…HE	2.17–2.36
					THR620…O18	OG1…HG	2.29–2.53
					THR622…O22	N…HN	2.39–2.56
					N30…PHE463	NH…O	2.17–2.34
					N24…THR465	NH…OG1	2.37–2.53
					O18…THR620	OH…OG1	2.08–2.27

**Table 5:** Inhibition zones to the selected antibiotics for pathogens belonging to both Gram-negative (GN) and Gram-positive (GP) classes such as *Aeromonas hydrophila* PAI-45 and PI-88 (GN), *Morganella morganii* PI-81(GN), *Bacillus cereus* ESN-09 (GP), and *Enterococcus lactis* CE-13 (GP) and *Enterococcus durans* CI-28 (GP). Inhibition areas are in mm, while R means “*resistant*”.

		BPN	OXN	APN	CBC	AZL	TRC
GN	*A. hydrophila* PAI-45	R	R	R	R	14.3	19.9
	*A. hydrophila* PI-88	R	R	14.2	20.7	14.5	12.3
	*M. morganii* PI-81	R	R	R	R	R	R
GP	*B. cereus* ESN-09	R	R	R	R	R	-
	*E. lactis* CE-13	14.7	R	22.7	14.2	18.7	-
	*E. durans* CI-28	16.3	R	24.0	16.1	20.1	-

## Data Availability

Not applicable.

## Supplementary Materials

The following supporting information can be downloaded at: https://www.mdpi.com/article/10.3390/ijms232012685/s1.
