# Supplementary material for: Cheminformatics Study on Structural and Bactericidal Activity of Latest Generation β-Lactams on Widespread Pathogens
Source: Int J Mol Sci. 2022 Oct 21;23(20):12685. doi: 10.3390/ijms232012685 (PMC9604271; doi:10.3390/ijms232012685)
Supplement: Supplementary file 1 [file ijms-23-12685-s001.zip › ijms-1958447-supplementary.pdf]

Supplementary Data

Cheminformatic study on structure and bactericidal activity of latest generation β-lactams on widespread pathogens

Ana Maria Raluca Gherman[1,2], Vasile Chiș[2] and Nicoleta Elena Dina[2]*

[1]*Department of Molecular and Biomolecular Physics, National Institute for R&D of Isotopic and Molecular Technologies, Donat 67-103, 400293 Cluj-Napoca, Romania*

[2]*Faculty of Physics, Babeș-Bolyai University, Kogălniceanu 1, 400084 Cluj-Napoca, Romania*

*Corresponding author at Department of Molecular and Biomolecular Physics, National Institute for R&D of Isotopic and Molecular Technologies, Donat 67-103, 400293 Cluj-Napoca, Romania

E-mail address: nicoleta.dina@itim-cj.ro (Nicoleta Elena Dina)

Table S1 – Assignment for the common FT-Raman bands (1064 nm excitation laser line) observed for BPN, OXN, APN, CBC, AZL as suggested by DFT calculations performed at B3LYP/6-311+G(2d,p) – harmonic (ω_H) and 6-31g(d) – anharmonic level (ω_A) of theory, in gas phase.

BPN	BPN ω_H	BPN ω_A	OXN	OXN ω_H	OXN ω_A	APN	APN ω_H	APN ω_A	CBN	CBC ω_H	CBC ω_A	AZL	AZL ω_H	AZL ω_A	Assignments
99sh (m)	77	91	107 (m)	-	-	-	-	-	117	-	-	122	-	-	τ(OCO) + τ(benzene ring)
130 (s)	112	125		105	113		-	-		-	-		-	-	τ(benzene ring) + δ(CO) (2-azetidinone ring) + δ(NH) + τ(CH$_3$) (side chain)
	-	-	-	-	-	118	116	112		82	66		-	-	β(CCC) (benzene ring and side chain)
	-	-	-	-	-	-	-	-		-	-		121	121	δ$_{OUT}$(ring) + δ$_{OUT}$(C=O) (2-azetidione ring)
	-	-	127 (w)	146	147	-	-	-		-	-		-	-	δ(NH) + τ(side chain ring) + ρ(benzene ring) + ω(CH$_3$) (side chain)
	-	-	-	147	-	-	-	-		-	-		-	-	τ(isoxazole ring) + ρ(benzene ring) + δ(NH) + δ(CO) (side chain)
	-	-	149 (m)	-	150	148	-	-		-	-		-	-	ρ(CH$_3$) (side chain) + ρ(benzene ring) + ρ(isoxazole ring)
	-	-	-	-	-	160	158	156		-	-		-	-	ρ(NH$_2$)
	-	-	171 (w)	164	166	174	172	175		-	-		-	-	ρ(NH$_2$) + τ(benzene ring) + ω(CH$_3$)
							175	265							ρ(NH$_2$)
180 (m)	177	185	187 (vw)	202	199	-	-	-		-	-	188	190	180	ρ(CH$_2$) + δ$_{OUT}$(NH) + δ$_{OUT}$(C=O) (ring and 2-azetidione ring) + δ$_{IN}$(OH)
208 (w)	188	196	211 (vw)	202	210	209	210	253	206	-	-	-	-	-	ρ(benzene ring) + τ(NH$_2$)
-	-	-		205	-	-	-	-	-	-	-	-	-	-	β(benzene ring; isoxazole ring)
-	-	-	233 (vw)	235	238	228	222	220	-	-	-	-	-	-	β(CNC) + ω(CH$_3$)
231 (w)	213	236	-	-	-	240	236	237	246	246	240	241	241	221	ρ(CH$_3$)
-	-	-	250 (vw)	214	245	251 (vw)	244	230	-	-	-	-	-	-	τ(CH$_3$)
-	-	-	-	264	265	-	-	-	-	-	-	-	-	-	τ(CH$_3$) + τ(ring 4) + β(benzene ring)
274 (m)	263	275	279 (vw)	283	286	271	273	270	280	287	283	-	-	-	ρ(CH$_3$)
292 sh (vw)	-	-	296 sh (vw)	285	289	291	301	303		290	291	294	298	291	ω(CH$_3$) + β(CCC)
320 (w)	324	325	-	-	-	317	318	314	317	305	300	-	-	-	ρ(CH$_3$) + ρ(OCO) + ρ$_{out\ of\ plane}$(thiazolidine ring)
-	-	-	319 (w)	328	334	-	-	-	-	-	-	-	-	-	β(NCC) + τ(CH$_3$) (penam core)
333 (w)	-	331	-	-	-	-	-	-	335	341	332	-	-	-	ρ(OCO) + ρ$_{in\ plane}$(benzene ring) + ρ(CH$_3$)
-	-	-	-	-	-	340	334	333	-	-	-	-	-	-	δ$_{OUT}$(CH) - out of plane deformation of benzene ring + β(CCN) + β(CCC) + ω(CH$_3$)
-	-	-	336 (w)	352	353	-	-	-	-	-	-	-	-	-	ν(CS) + β(benzene ring; isoxazole ring) + ρ(CH$_3$) (penam core)
-	-	-	-	-	-	351	353	352	-	-	-	-	-	-	β(CCC) + ω(CH$_3$)

Supplementary Data

360 (w)	349	353	-	-	-	360	364	365	361	363	359	360	366	362	β(CCC) from CH_3-C-CH_3
-	361	368	368 (vw)	379	386	-	-	-	-	-	-	-	-	-	β(CCC) + ω(CH_3) + ρ(OCO) + ω(benzene ring) + τ(isoxazole ring)
-	-	-	-	-	-	388	378	376	383	398	389	387	392	383	β(CNC) + β(CCO) (penam core)
-	-	-	388 (vw)	392	398	-	-	-	-	-	-	-	-	-	β(side chain; isoxazole ring) + ρ(benzene ring) + ω(CH_3)
392 (vw)	382	390	-	-	-	-	-	-	-	-	-	-	-	-	β(CCN) + β(CCO) + δ(OH) + δ(NH)
402 (vw)	-	-	406 (vw)	405	415	409	-	-	405	414	407	409	410	407	δ_{IN}(C=O) (benzene ringring and chain)
412	398	415	-	-	-	-	-	-	-	-	-	-	-	-	δ(CH) out of plane – out of plane deformation of benzene ring
-	-	-	-	-	-	423	426	421	-	-	-	425	-	-	β(NCC) + δ_{OUT}(CH) (benzene ring)
448 (vw)	448	455	443 (vw)	422	424	-	-	-	-	-	-	-	-	-	β(CCC) + ω(CH_3) (side chain)
468 (m)	477	476	-	-	-	465	462	460	-	-	-	465	491	463	δ_{OUT}(NH) (imidazolidine ring)
478sh (w)	492	487	477 (vw)	461	463	-	-	-	-	-	-	479	491	478	δ_{OUT}(NH) (imidazolidine ring)
-	-	-	-	-	-	483	484	479	480	494	484	-	-	-	β(NCS) + δ_{OUT}(CH) + δ_{OUT}(NH) + δ_{OUT}(OH)
-	-	-	492 (vw)	492(vw)	481	-	-	-	-	-	-	-	-	-	δ(NH)
-	-	-	-	-	-	498	493	491	-	-	-	-	-	-	β(CNS) + β(OCN) + δ(CH) + δ(NH)
514 (vw)	522	529	-	-	-	-	-	-	-	-	-	510	-	-	β(CCC) + ω(CH_3) + β(CCN) + δ(NH) + δ(CH) (2-azetidinone ring
-	-	-	522 (w)	501	505	522	531	526	525	522	521	-	-	-	δ_{OUT}(OH) + β(CCC) + δ_{OUT}(CH) + δ_{OUT}(NH) + ω(CH_3)
534 (vw)	-	-	-	-	-	-	-	-	-	-	-	532	537	529	δ_{OUT}(benzene ring)
-	-	-	541 (vw)	523	-	--	-	-	-	-	-	-	-	-	β(CCN) – in plane deformation of thiazolidine ring + δ(CH) (2-azetidinone ring) + δ(NH) + β(CCC) + ω(CH_3)
-	-	-		558	-	-	-	-	-	-	-	-	-	-	δ(CH) out of plane (benzene ring) + ν(CS) – in plane deformation of thiazolidine ring + β(CCC) (penam core)
571sh (m)	556	569	579 (w)	561	575	-	-	-	575	571	559	575	571	558	β(CCC) (thiazolidine ring)
582 (m)	574	586		-	-	-	-	-	-	-	-	-	-	-	β(CCN) + ν(CS) – in plane deformation of thiazolidine ring + δ(CH) (2-azetidinone and benzene ring) + δ(NH)
-	-	-	-	-	-	588	570	558	589	598	586	-	-	-	β(OCC) + δ_{OUT}(CH) + δ_{OUT}(OH) + δ_{OUT}(NH)
-	-	-	-	-	-	-	-	-	-	-	-	594	592	580	δ_{OUT}(CH) out of phase (2-azetidione ring) + ρ(CCC) (penam core) + δ_{IN}(NH) (side chain)
602 sh (vw)	606	617	616 (w)	592	603	601	602	594	607	-	-	-	-	-	β(NCS) – in plane deformation of thiazolidine ring + δ(CH) + δ(NH)
-	-	-	629 (vw)	626	633	615	-	-	617	631	623	617	634	625	δ_{OUT}(NH) + in plane deformation of benzene ring + δ_{IN}(imidazolidine ring)
621 (w)	628	630	-	-	-	-	-	-	-	-	-	-	-	-	ω(CCC) – out of plane deformation of benzene ring + δ(CH) out of plane (benzene ring) + ν(CS) – in plane deformation of thiazolidine ring + δ(CH) (2-azetidinone ring)
-	-	638	-	-	-	635	650	641	-	-	-	-			β(NCC) + δ(CH) (penam core)
-	-	-	-	-	-		-	-	-	-	-	641	641	626	δ_{OUT}(NH) (benzene ring)
-	-	-	648 (w)	642	648	-	-	-	-	-	-	-		-	out of plane deformation of isoxazole ring + ρ(CH_3) (side chain)
647 (vw)	647	661		643	654	-	-	-	-	-	-	-	-	-	β(CNC) – in plane deformation of thiazolidine ring + δ(CH) (penam core) + β(NOC) – out of plane deformation of isoxazole ring + ω(CH_3) (side chain)
661 (w)	656	668	-	-	-	-	-	-	666	663	643	661	664	654	δ_{OUT}(NH) + δ_{OUT}(CH) + δ_{OUT}(OH)
-	-	-	656 sh	653	665	670	666	656	-	-	-	-	-	-	ν(CS) (in plane deformation of thiazolidine ring) + β(OCO) + δ(OH) + δ(CH) + δ(NH)
-	-	-		661	670	-	-	-	-	-	-	-	-	-	β(CCC) – in plane deformation of benzene ring + δ(CH) in plane (benzene ring) + in plane deformation of isoxazole ring + ν(CC)
-	-	-	688sh (vw)	703	-	-	-	-	-	-	-	-	-	-	τ(CCC) – out of plane deformation of benzene ring and isoxazole ring)

-	-	-	-	-	-	695	715	700	-	-	-	-	-	-	δ_{OUT}(CH) - of benzene ring
703 (vw)	-	-	702 (vw)	700	-	-	-	-	-	-	-	-			δ(CH) (benzene ring)
721 (w)	727	743	-	-	-	723	-	-	719	741	726	714	719	704	δ_{OUT}(CH) (benzene ring) + δ_{OUT}(NH) + ρ(CH_2) (imidazolidine ring)
-	-	-	-	-	-	730	742	726	-	-	-	734	743	728	β(CCC) + τ(CH_3) + δ_{OUT}(NH) (side chain)
-	-	-	734 (vw)	726	736	-	-	-	-	-	-	-	-	-	β(CCN) – in plane deformation of 2-azetidinone ring + δ(CH) (2-azetidinone ring) + ν(CC) – in plane deformation of isoxazole ring + β(CCC) – in plane deformation of benzene ring
748 (w)	742	756	747 (vw)	727	745	-	-	-	746	767	751	-	-	-	δ_{OUT}(CH) (benzene ring) + β(OCO) + δ(CH)
-	-	-	-	-	-	752	754	744	-	-	-	-	-	-	β(CCN)+ δ(CH) + δ(OH) (penam core)
762 (vw)	765	777	766 (vw)	754	763	-	769	760	767	800	766	-	-	-	δ_{OUT}(CH) (benzene ring) + β(OCN)
776 (vw)	779	-	777 (vw)	766	784	-	-	-	-	-	-	-	-	-	δ(CH) (benzene ring) + ω(CCN) (side chain)
-	-	-	-	-	-	780	-	779	-	-	-	786	803	789	β(CCN) + β(CNC) (side chain)
-	-	-	793sh (vw)	787	803	-	-	-	-	-	-	-	-	-	δ(CH) (benzene ring) + ω(CCC) – out of plane deformation of isoxazole ring + ω(CH_3) (side chain)
807 (vw)	800	806	-	776	807	802	806	803	803	808	786	-	-	-	δ(OH) + δ(CH) + δ(NH) + β(CNC) + β(OCO)
-	-	-	-	-	-	-	-	-	-	-	-	813	807	792	in plane deformation of 2-azetidione ring + δ_{OUT}(OH) + δ_{OUT}(CH) (thiazolidine ring)
-	-	-	813 (vw)	805	815	-	-	-	-	-	-	-	-	-	
-	-	-		808	825	830	838	831	834	852	813	-	-	-	δ_{OUT}(CH) (benzene ring) (+ δ_{OUT}(OH))
839 (w)	-	849	845(vw)	846	862	-	-	-	-			-	-	-	δ(CH) (benzene ring)
-	-	-	-	-	-	847	858	856	847	877	856	850	888	866	δ_{OUT}(OH)
873 (w)	-	862	-	-	-	873	873	858	876	887	869	870	-	-	δ(OH) + β(CNC) + in plane deformation of benzene ring
895 (w)	-	879	-	-	-	-	-	-	-	-	-	-	-	-	δ(OH)
-	-	-	-	-	-	-	-	-	-	-	-	899	914	902	β(CNC)
-	-	-	894 (w)	887	900	910	903	903	891 sh	887	910	-	-	-	δ_{OUT}(CH) (benzene ring) + δ(OH)
-	-	-	908 (vw)	921	917	-	-	-	-	-	-	-	-	-	ν(ON) – in plane deformation of isoxazole ring + ω(CH_3) (side chain) + δ(CH) (benzene ring)
910 (vw)	910	-	-	-	-	926(vw)	925	923	-	-	-	913	930;933	913;921	δ_{OUT}(CH) (benzene ring); β(CNC) (thiazolidine ring)
919 (vw)	919	925	920 (vw)	920	933	-	-	-	920	933	922	-	-	-	β(CNC) + ν_{as}(OCO) + ω(CH_3)
933 (vw)	937	934	-	-	-	-	-	-	-	-	-	-	-	-	β(CNC) + ν(CC) (2-azetidinone ring) +ν_{sym}(OCO) + δ(CH)
945 (vw)	948	950	944 (vw)	950	958	-	-	-	-	-	-	-	-	-	(CC) + δ(CH) (2-azetidinone ring) + ω(CH_3) (penam core)
-	-	-	-	-		951	952	950	948	959	945	948	951	933	ω(CH_3)
960 (vw)	982	964	-	-	-	961	964	975	-	-	-	958	962	947	ω(CH_3) + ν(CC) + δ(NH) (imidazolidine ring)
-	-	-	-	-	-	-	-	-	957	982	953	-	-	-	δ_{OUT}(CH) (benzene ring)
983sh (vw)	1016	1016	982sh (w)	993	968	982	-	-	-	-	-	988 sh	-	-	δ(CH) (benzene ring)
-	-	-	-	997	976	993	971	990	-	-	-	-	-	-	β(CCC) – in plane deformation of benzene ring
1002 (vs)	1005	989	1002 (vs)	1006	987	1004	988	1009	1004	989	1000	1003	989	1008	in plane deformation of benzene ring
-	-	-	-	-	-	-	993	980	-	982	1008	-	994	984	δ_{IN}(CH) out of phase (2-azetidinone ring)
1029 (m)	-	-	1026 (vw)	1039	1013	1027	997; 1014	1032; 1032	1032	1017	1039; 1045	1030	1017	1042	δ_{IN}(CH) out of phase (benzene ring)
-	-	-	1035 sh (vw)	1045	1018	1037	1015	1044	-	-	-	-	-	-	β(CCC) + δ(CH) (benzene ring) + ω(CH_3)
-	1042	1059	-	-	-	1071	1073	1095	1076	1058	1078	1070	1073	1071	ν(CN) (β-lactam ring)
1090 (vw)	1150	1109	-	-	-	-	-	-	1089	-	-	1090	1093	1094	δ_{IN}(CH) out of phase (benzene ring)
-	-	-	-	-	-	-	-	-	1098	1070	1114	-	-	-	in plane def of benzene ring + δ_{IN}(CH)
1122 (w)	1168	1129	1113sh(vw)	1157	1130	-	-	-	-	-	-	-	-	-	ν(CC) + δ(CH)
-	-	-	-	-	-	1119	1124	1149	-	-	-	-	-	-	ν(CN) + δ(NH) + δ(CH) + τ(NH_2)/ ω(CH_3) + ν(CC) + δ(CH)

Supplementary Data

-	-	-	-	-	-	-	-	-	-	-	-	1125	1109	1128	ν(NCN) + τ(CH_2) – in plane deformation of imidazolidine ring
-	-	-	1126 (vw)	1197	1147	-	-	-	1126	1109	1128	-	-	-	ω(CCC)+ τ(CH_3) + δ_{OUT}(CH)
-	-	-		1183	1148	-	-	-	-	-	-	-	-	-	δ(CH) (2-azetidinone ring)
1156 (w)	1183	1145	1148sh (w)	1209	1167	-	-	-	-	-	-	-	-	-	δ(CH) in plane (benzene ring)
-	1189	1147	-	-	-	1156	1151	1156	1156	1144	1174	1158	1110	1132	δ_{OUT}(CH) out of phase (2-azetidinone ring)
-	-	1155	-	-	-	-	-	-	-	1146	1177		1129	1151	δ_{OUT}(CH) out of phase (2-azetidinone ring)
-	-	-	-	-	-	-	-	-	-	-			1135	1151	β(CNC) (side chain)
-	-	-	1163 (w)	1201	1171	-	-	-	-	-		-	-	-	ν(CN) + δ(CH) (2-azetidinone ring)
1176 (w)	1205	1183	-	-	-	1178	1176	1160	-	-		1175	-	-	ν(CC) + δ(CH) + δ(NH) + ω(CH_3)
-	-	-	1181 (vw)	1183	1199	-	-	-	1180	1159	1180	-	-	-	ν(CN) + δ(CH) (2-azetidinone)
-	-	-	-	-	-	1186	1178	1175	-	-	-	1186	1170	1190	δ_{IN}(CH) out of phase (benzene ring)
1201 (w)	1220	1201	-	-	-	1197	1192	1192	-	-		-	-	-	δ_{IN}(CH) in plane (2-azetidinone ring) + ν(CN) + δ(NH)
-	-	-	-	-	-	-	-	-	-	-	-	1211	1193	1216	δ_{OUT}(CH) out phase (2-azetidinone ring) + δ(NH) + δ(CH) (side chain)
-	-	-	-	-	-	1217	1200	1209	-	-	-	-	-	-	δ_{OUT}(CH) in plane (2-azetidinone ring) + ν(CN) + δ(NH)
-	-	-	1220 (vw)	1222	1242	-	-	-	-	-	-	1223	1204	1227	δ(CH) in phase (2-azetidinone ring) + δ_{IN}(NH) + δ_{IN}(CH) (side chain)
1232sh (w)	-	1229	-	-	-	1233	1228	1230	-	-	-	-	-	-	δ_{IN}(CH) out of plane (2-azetidinone ring)
1245 (w)	1257	1241	-	-	-	1249	1241	1277	-	-	-	-	-	-	ν(OH) + ν(CC) + δ(OH)
-	-	-	1251sh (vw)	1260	1262	1255	1266	1265	1252	1229	1250	-	-	-	δ(CH) + δ(NH) + δ(OH)
-	-	-	1264 (w)	1267	1272	-	-	-	-	-	-	1261	1229	1261	δ_{OUT}(CH) (penam core)
-	-	-		1273	1274	-	-	-	-	-	-	-	-	-	ν(CC) + ν(CN) + δ(CH) + δ(NH) + δ(OH) (penam core)
-	-	-	-	-	-	-	-	-	-	-	-		1239	1266	ω(CH_2) (imidazolidine ring)
1292(m)	1307	1283	-	-	-	-	-	-	1297	1271	1289	-	-	-	δ_{OUT}(CH) (thiazolidine ring + beta-lactam ring)
-	-	-	-	-	-	-	-	-	-	1273	1290	-	-	-	δ_{OUT}(CH) (thiazolidine ring)
1304sh(vw)	1295	1270	1308 (vw)	1300	1312	-	-	-	-	-	-	-	-	-	ν(CN) + ν(CO) – in plane deformation of isoxazole ring + ν(CC) – in plane deformation of benzene ring + δ(CH) (benzene ring) + δ(NH) + δ(CH) (penam core)
-	-	-	1317 (vw)	1303	1317	1315	1318	1308	1319	1293	1329	1317	1278	1311	δ_{IN}(CH) + δ_{IN}(NH) (side chain) + δ(CH) (benzene ring)
1328(w)	1327	1306	-	-	-	-	-	-	-	-	-	-	-	-	δ(CH) + δ(NH) + β(CNC) + ω(CH_2)
-	-	-	1353 (w)	1343	1355	1349	1350	1339	-	-	-	-	-	-	ν(CC) + δ(CH) in plane (benzene ring) + τ(NH_2)
-	-	-	-	-	-	-	1354	1343	-	-	-	-	-	-	ν(CN) + δ(CH) (2-azetidinone)
-	-	-	-	-	-	1363	1367	1361	-	-	-	-	-	-	β(CH_3) out of phase
1370(vw)	1377	1355	1371 (vw)	1356	1364	-	-	-	1372	1355	1373	1369	1342	1365	δ(CH) (benzene ring) + δ(CH) (side chain)
-	-	-	-	-	-	1382	1385	1380	1386	1355	1389	-	-	-	δ_{OUT}(CH) + δ_{IN}(OH) (penam core)
-	-	-	1384 (vw)	1388	1382	-	-	-	-	-	-	-	-	-	(CH_3) + ν(CC) (isoxazole ring) + δ(CH) in plane (benzene ring)
-	-	-	-	-	1385	-	-	-	-	-	-	-	-	-	δ(OH) (penam core) + β(CH_3)
-	-	-	1390 (vw)	1397	1390	1392	1389	1398	-	-	-	1397	1374	1399	ω(CH_2) + δ_{IN}(NH) (imidazolidine ring)
1419 (w)	1403	1412	1418sh (vw)	-	-	-	-	-	-	-	-	-	-	-	β(CH_2)
1436 (w)	1424	1435	1433 sh	-	-	1435	1435	1410	1436	1435	1460	1437	1434	1454	β_{as}(CH_3) out of phase
-	-	-	1444 (m)	1443	1415	-	-	-	-	-	-	-	-	-	β(CH_3) (side chain)
-	-	-	-	1458	1425	-	-	-	-	-	-	-	-	-	ν(CC) + δ(CH) in plane (benzene ring) + ν(C=N) + β(CH_3) (isoxazole ring)
1452 (vw)	1449	1457	1459	-	-	1456	1457	1446	1457	1456	1488	1458	1442	1476	δ(CH) (benzene ring + side chain)
1468 (vw)	1475	1481	-	-	-	-	1457	-	-	1457	1489	-	1456	1477	β_{sym}(CH_3) in phase
1499 (vw)	1499	1483	1471 (m)	1483	1450	-	-	-	-	-	-	-	-	-	ν(C=N) + ν(CC) (ring 4) + ν(CCC) + δ(CH) (benzene ring) + β(CH_3) (side chain)
-	-	-	-	-	-	-	-	-	-	-	-	1491	1457	1482	β_{as}(CH_3) in phase
-	-	-	-	-	-		-	-	-	-	-		1476	1508	β(CH_2) in phase
-	-	-	1516 (w)	1540	1490	1512	1477	1530	-	-	-	-	-	-	ν(CN) + δ(NH)
-	-	-		1526	1490	-	-	-	-	-	-	1532	1512	1524	ν(CH) (chain) + δ_{IN}(NH) (chain and side chain)
-	-	-	1556 (vw)	1588	1553	-			-	-	-	-	-	-	ν(C=C) + δ(CH) in plane + δ(NH) + ω(CH_3) (isoxazole ring and benzene

Supplementary Data

															ring)
1582 (m)	1600	1568	1578 (w)	1605	1569	1585	1570	1605	1582	1571	1606	1585	1571	1606	ν(CC) + δ_{IN}(CH) (benzene ring)
1600 (m)	1620	1588	1606 (vs)	1621	1588	1602	1588	1623	1600	1584	1619	1602	1591	1626	ν(CC) + δ_{IN}(CH) (benzene ring)
-	-	-	-	-	-	1638	1613	1628	-	-	-	-	-	-	β(NH_2)
1638 (vw)	1752	1679	1649 (w)	1735	1652	-	-	-	1666	1681	1743	1660	1631	1687	ν(C=O) + δ_{IN}(NH) (chain and benzene ring)
-	-	-	-	-	-	-	-	-	-	-	-	1683	1695	1769	ν(C=O) + δ_{IN}(NH) + δ_{IN}(CH) (side chain)
-	-	-	-	-	-	1693	1680	1756	-	-	-	-	-	-	ν(C=O) (side chain)
1700 (w)	1784	1722	-	-	-	-	-	-	-	-	-	-	-	-	ν(C=O) + δ(OH)
1775 (w)	1808	1745	1755 (vw)	1786	1720	1764	-	-	1766	1717	1778	-	1742	1814	ν(C=O) (on penam core) + δ_{IN}(OH) + δ_{IN}(CH) (thiazolidine ring)
			-	-	-	-	1723	1797		1721	1781				ν(C=O) + δ_{IN}(OH) (penam core)
			1772sh(vw)	1801	1744	-	1745	1822		1744	1812				ν(C=O) + δ_{IN}(OH) (penam core)
-	-	-	-	-	-	2714	-	-	-	-	-	-	-	-	-
2727(vw)	-	-	2729(vw)	-	-	2729	-	-	2731	-	-	2726	-	-	-
2738sh(vw)	-	-	2749sh(vw)	-	-	2757	-	-	-	-	-	-	-	-	-
2769(vw)	-	-	2771 (vw)	-	-	-	-	-	2771	-	-	2770	-	-	-
2785sh(vw)	-	-	-	-	-	-	-	-	-	-	-	-	-	-	-
2823(vw)	-	-	-	-	-	-	-	-	-	-	-	-	-	-	-
2859(vw)	-	-	2862sh (vw)	-	-	-	-	-	2861	-	-	2861	-	-	-
-	-	-	-	-	-	2872	-	-	2873	-	-	-	-	-	-
2883sh(vw)	-	-	2883sh (vw)	-	-	-	-	-	2883	-	-	-	-	-	-
2896sh(w)	-	-	-	-	-	-	-	-	-	-	-	-	-	-	-
-	-	-	2906sh (vw)	-	-	2902	2899	2884	2904	-	-	-	2916	2885	$\nu_{sym}(CH_2)$ in phase
2918(s)	2905	2932	-	-	-	-	-	-	-	-	-	2914	2931	2916	$\nu_{sym}(CH_3)$ out of phase
-	-	-	2930 (m)	2936	2932	2931	-	-	2932	2938	2953	2933	2937	2949	$\nu_{sym}(CH_3)$ in phase
2936(m)	2919	2938	2937sh	2923	2938	2940	2932	2922	-	-	-	-	-	-	$\nu_{sym}(CH_3)$ out of phase
-	-	-		2963	2939	-	-	-	-	-	-	-	-	-	$\nu_{sym}(CH_3)$ (isoxazole ring)
2957(s)	2958	2951	2952 (w)	2962	2976	2954	-	-	-	-	-	-	-	-	ν(CH) (thiazolidine ring)
-	-	-	2971 (w)	2964	2993	-	-	-	2970	2998	2989	-	-	-	$\nu_{as}(CH_3)$ (side chain)
-	-	-	-	2976	2994	2975	2977	2962	-	-	-	2975	2994	2987	$\nu_{as}(CH_3)$ out of phase
2979(s)	2980	2977	-	2990	2995	-	-	-	2980 sh	-		-	-	-	$\nu_{as}(CH_3)$ (penam core) + ν_{OUT}(CH) (2-azetidinone ring)
-	-	2984	-	2983	2999	2985	2993	2972	-	-		-	-	-	ν(CH) out of phase (2-azetidinone ring)
-	-	2989	2986 (w)	2995	3001	-	-	-	-	-	-	-	-	-	$\nu_{as}(CH_3)$ (penam core)
3002(vw)	3017	2994	3010 (w)	3004	3009	3009	3009	3004	-	-	-	-	-	-	$\nu_{as}(CH_3)$ out phase
3031 sh	-	3009	3029sh (vw)	-	-	3031			3029	3060	3024	-	-	-	ν_{as}(CH) out of phase (benzene ring)
3041sh (m)	3048	3052	-	-	-	3048	3047	3037	-	-	-	-	-	-	ν_{as}(CH) in phase (benzene ring)
-	-	3054	3055sh (w)	3048	3049	-			-	-	-	-	-	-	$\nu_{as}(CH_3)$ (side chain)
3060(m)	3074	3073	3066 (m)	3064	3062	3061 (m)	3084	3076	3061 (m)	3087	3082	3061	3087	3087	ν(CH) (benzene ring) in phase
3163 (vw)	3090	3186	3211 (vw)	3118	3192	3169	3189	3108	-	-	-	-	-	-	ν(OH)
-	-	-	-	-	-	3333	3369	3318	-	-	-	-	-	-	$\nu_{sym}(NH_2)$
3355(w)	3417	3477	-	-	-	-	3441	3351	-	-	-	-	-	-	$\nu_{as}(NH_2)$
-	-	-	-	3419	3482		3468	3399		-	-	-	-	-	ν(NH)

ν – stretching; β – bending; δ – out of plane bending; ρ – rocking; ω – asymmetric stretching; τ – twisting; HB – hydrogen bonding

Supplementary Data

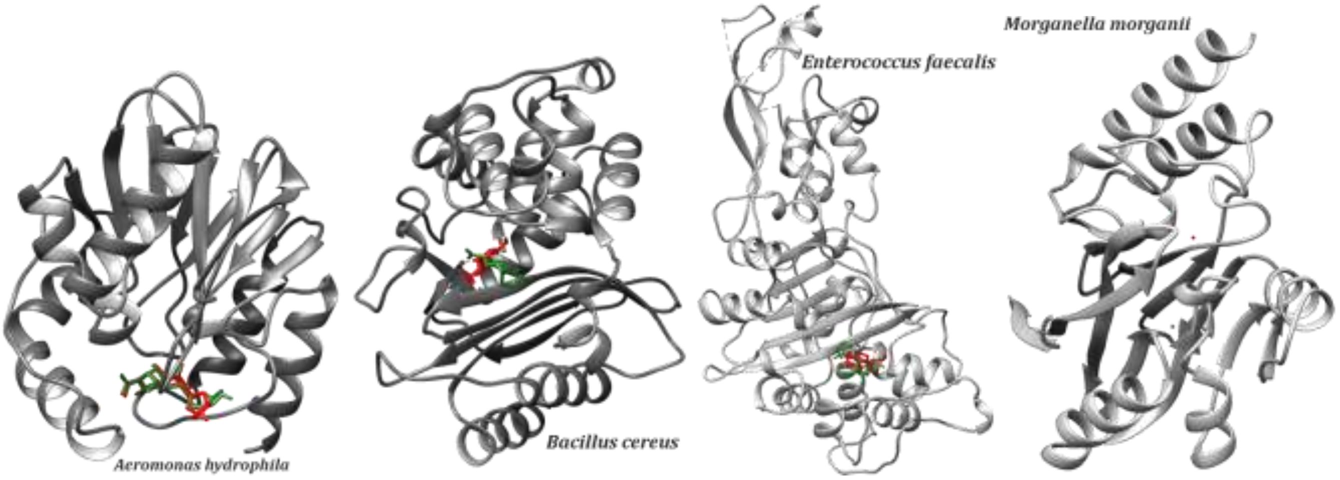

Figure S1 - Docking position (green) of the original ligands overlapped to their initial position (red) as found in the complex with the selected receptors in crystal structure form – *A. hydrophila* – chain A of the zinc carbapenemase CphA (PDB id: 1x8i) in complex with biapenem, *M. morganii* – chain A from metallo-beta-lactamase IMP-27 (PDB id: 6l3s) in complex with Zn^{2+}, *B. cereus* – chain A from class A beta-lactamase (PDB id: 6w33) in complex with clavulanate, and *E. faecalis* – chain A of penicillin-binding protein 4 PDB4 (PDB id: 6mkh) in complex with imipenem.

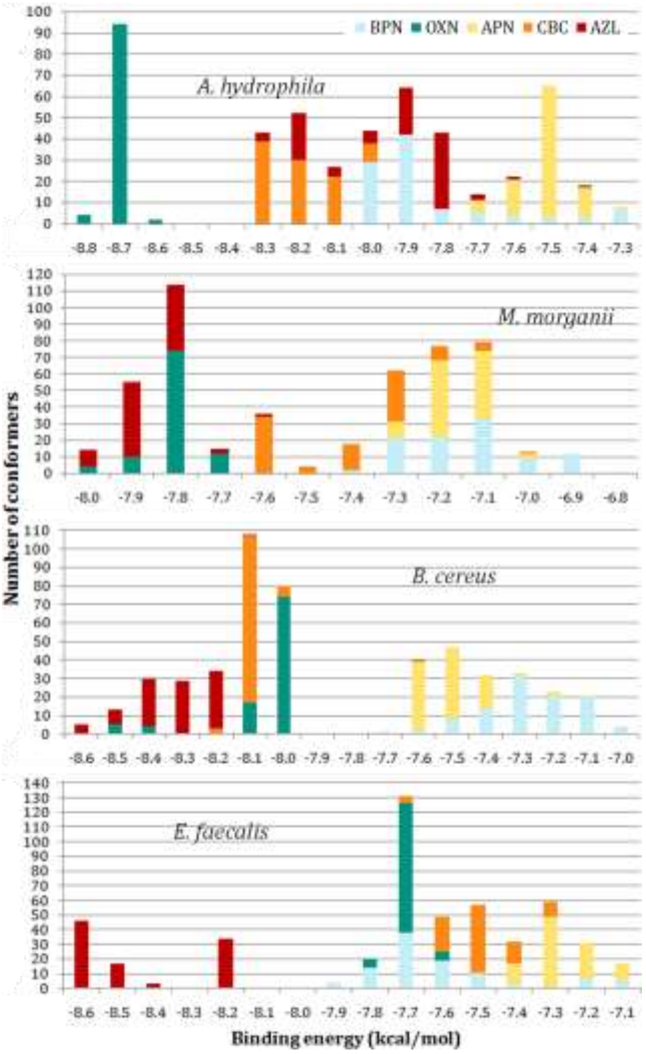

Figure S2 – Number of conformers scoring the lowest values of the binding energy (kcal/mol) for each of the hundred runs of the molecular docking for all twenty ligand-receptor systems considered, with *A. hydrophila*, *M. morganii, B. cereus, and E. faecalis* as receptors and benzylpenicillin (BPN – light blue), oxacillin (OXN - petrol), ampicillin (APN - yellow), carbenicillin (CBC - orange), and azlocillin (AZL - red), as ligands.

Supplementary Data

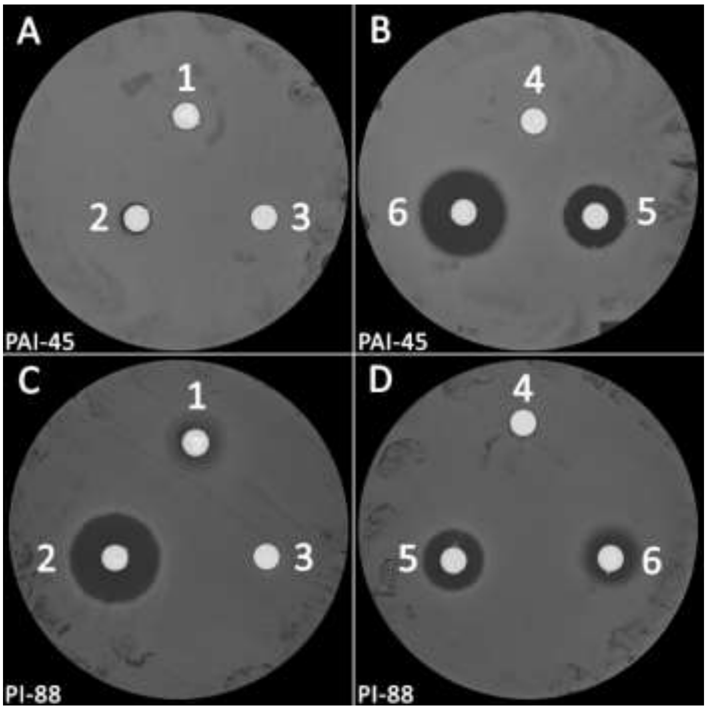

Figure S3 – Optical images representing sensitivity tests to six selected antibiotics of the Gram-negative *A. hydrophila* specie PAI-45 (A and B) and PI-88 (C and D). Both resistance and sensitivity to the antibiotics (1 – ampicillin, 2 – carbenicillin, 3 – oxacillin, 4 – penicillin G, 5 – azlocillin, and 6 - tetracyclin) can be observed.

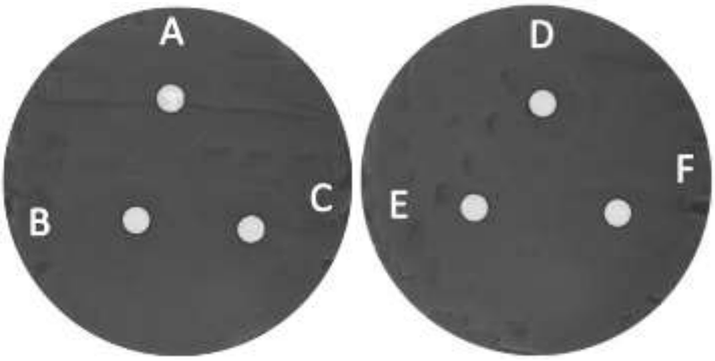

Figure S4 – Optical images representing sensitivity tests to six selected antibiotics of the Gram-negative *M. morganii* PI-81. The pathogen presented resistivity to all six antibiotics (A – ampicillin, B – carbenicillin, C – oxacillin, D – penicillin G, E – azlocillin, and F - tetracycline).

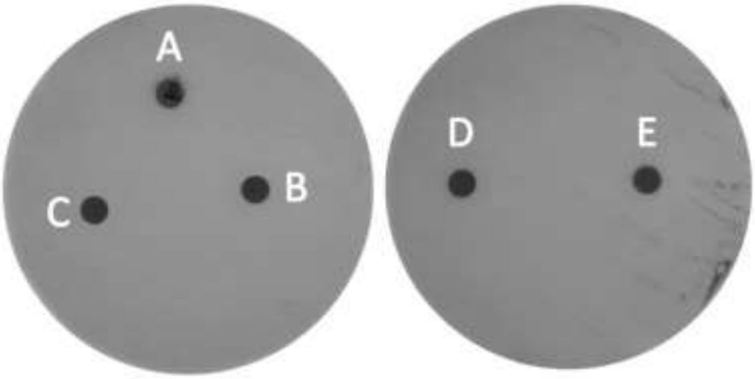

Figure S5 – Optical images representing the sensitivity tests to five antibiotics of Gram-positive pathogen *B. cereus* ESN-09, which presented resistance to all five antibiotics (A – ampicillin, B – carbenicillin, C- oxacillin, D – azlocillin, and E – penicillin G).

Supplementary Data

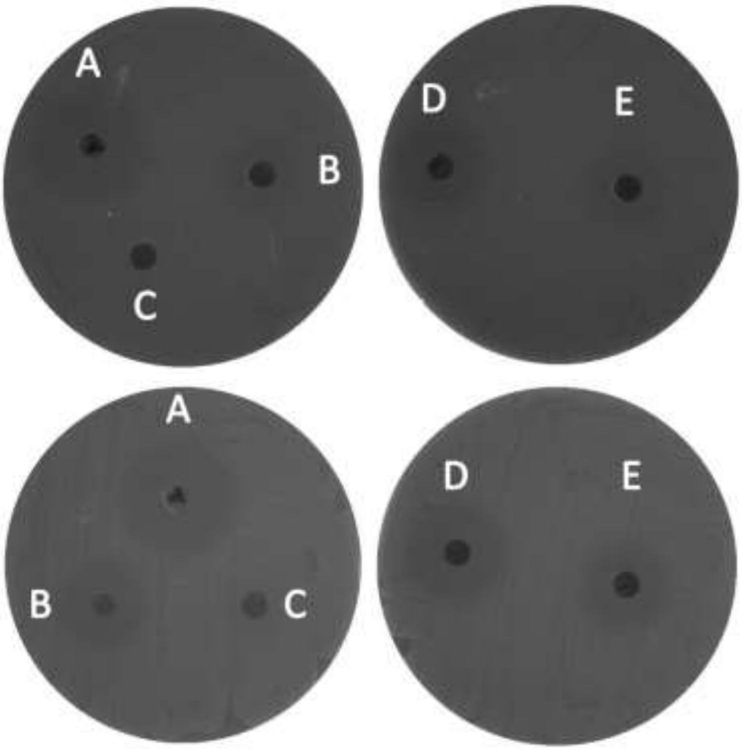

Figure S6 – Optical images picturing sensitivity tests to five antibiotics (A – ampicillin, B – carbenicillin, C- oxacillin, D – azlocillin, and E – penicillin G) of Gram-positive *E. lactis* CE-13 (top) and *E. durans* CI-28 (bottom) which show resistance to oxacillin, while being sensitive to the othe four considered antibiotics.

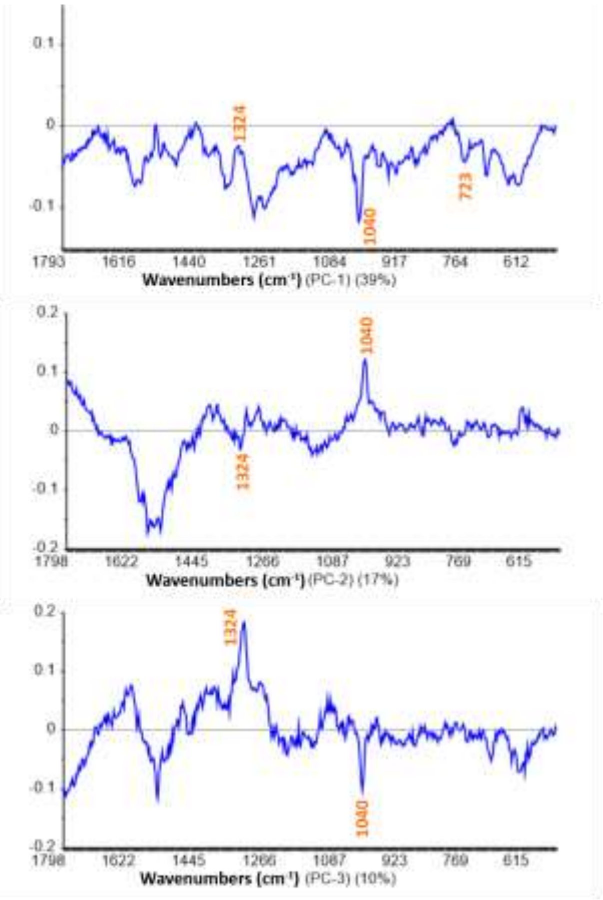

Figure S7 – Loadings plots for PC-1, PC-2, and PC-3 in the PCA performed on the full spectral range of the database containing same day samples. SERS marker bands with the greatest scores on the loadings plots are marked in orange for each PC.
